# M^6^A Modifier-Mediated Methylation Characterized by Diverse Prognosis, Tumor Microenvironment, and Immunotherapy Response in Hepatocellular Carcinoma

**DOI:** 10.1155/2022/2513813

**Published:** 2022-08-16

**Authors:** Fei Liu, Xinyue Zhang, Ziyu Liu, Weiye Cai, Chao Song, Yan Jiang, Ji Yin, Zongchao Liu, Chenyi Huang

**Affiliations:** ^1^Department of Orthopedics, The Affiliated Traditional Chinese Medicine Hospital of Southwest Medical University, Luzhou 646000, Sichuan, China; ^2^College of Integrated Chinese and Western Medicine, The Affiliated Traditional Chinese Medicine Hospital of Southwest Medical University, Luzhou 646000, Sichuan, China; ^3^Center for Phenomics of Traditional Chinese Medicine, The Affiliated Traditional Chinese Medicine Hospital of Southwest Medical University, Luzhou 646000, Sichuan, China; ^4^Department of Otorhinolaryngology, The Affiliated Traditional Chinese Medicine Hospital of Southwest Medical University, Luzhou 646000, Sichuan, China

## Abstract

**Objective:**

Emerging evidence highlights the clinical implications of N^6^-methyladenosine (m^6^A) modification in HCC. Yet, the roles of m^6^A modification in modulating cancer immunity and shaping tumor microenvironment (TME) are undefined in hepatocellular carcinoma (HCC).

**Methods:**

Here, m^6^A modification classification was determined for HCC through 23 m^6^A modifier levels by employing consensus clustering approach. Prognosis analysis was presented for comparing the differences in survival outcomes. The ssGSEA and ESTIMATE approaches were adopted for evaluating the abundances of tumor-infiltrating immune cell populations. The m^6^A scoring system was computed for reflecting m^6^A modification classification via PCA algorithm.

**Results:**

Three m^6^A modifier-mediated modification patterns were established among HCC specimens, which were characterized by different prognosis, signaling pathways, and TME features. After extracting m^6^A phenotype-associated DEGs, we determined m^6^A scores in individual HCC and stratified patients into high- and low-score groups. Patients with low m6A score displayed the survival advantage and higher sensitivity to gemcitabine. Moreover, those with low m^6^A score possessed the better anti-PD-1/PD-L1 therapeutic response in the IMvigor210 immunotherapy cohort.

**Conclusion:**

Our findings highlighted that m^6^A modification exerted a nonnegligible role in remodeling diverse and complex TME. Quantification of the m^6^A modification patterns of individual HCC may enhance the comprehension of TME features and facilitate immunotherapeutic plans.

## 1. Introduction

Hepatocellular carcinoma (HCC) represents a complex neoplasm with multiple etiologies, comprising 75% to 85% of liver cancer cases [1]. Over 1 million HCC patients will die from HCC in 2030, as estimated by the World Health Organization [2]. Early-stage HCC patients suitably receive curative therapy including resection, ablation, and transplantation, with expected 5-year survival rate up to 60% to 80% [3]. Nevertheless, less than 20% patients are eligible for curative therapy [4]. Intermediate-stage patients usually experience locoregional therapy [5]. Meanwhile, systemic therapy is reserved for advanced patients. For instance, sorafenib is the first systemic agent with efficacy for advanced HCC [6]. In recent years, immunotherapy like antiprogrammed cell death-1 (anti-PD-1), anti-PD-1 ligand (anti-PD-L1), and anti-cytotoxic T-lymphocyte antigen-4 (anti-CTLA-4) that may activate the host's natural defense system, and identify and eliminate the tumor cells [7], have emerged as a prospective alternative treatment strategy against advanced HCC with durable responses [8]. Despite this, only a minority of patients benefit from the immunotherapy [9]. Hence, it urgently demands novel therapeutic predictors for identifying the ideal HCC subgroups for immunotherapy.

N^6^-methyladenosine (m^6^A) is the most abundant messenger RNA (mRNA) modification that occurs in humans, occupying 0.1% to 0.4% total adenosine residues [10]. The m^6^A modification represents a dynamic reversible process in humans [11]. Hence, exploring the regulatory genes may assist to uncover the roles and mechanisms of m^6^A modification at posttranscriptional levels. Emerging evidences have confirmed that deregulation and genetic alterations of m^6^A modifiers contribute to HCC initiation and progression [12]. For instance, m^6^A reader YTHDF1 accelerates HCC progression via inducing FZD5 mRNA translation with an m^6^A-dependent manner [13]. M^6^A eraser ALKBH5 inhibits malignancy of HCC through m^6^A-dependent epigenetic suppression of LYPD1 [14]. M^6^A writer KIAA1429 facilitates migration and invasion of HCC through elevating m^6^A-mediated ID2 level [15]. HCC progression represents a multistep event, comprising the genetic and epigenetic alterations within tumor cells and the surrounding tumor microenvironment (TME) [16]. Cancer cells elicit various biological behavior alterations via the direct or indirect interplay with TME [17]. The in-depth comprehending of the diverse and complex TME may reveal its key roles in tumor development, immune escape, and immunotherapeutic responsiveness [18]. Emerging evidence suggests that TME is specially correlated to m^6^A modification [19]. For instance, m^6^A eraser ALKBH5 may enhance the effects of anti-PD-1 agent through modulating lactate accumulation along with immunosuppressive cell populations in the TME [20]. Inhibiting m^6^A writers METTL3/14 may increase the responsiveness to anti-PD-1 therapy [21]. Nevertheless, above findings are limited to one or two m^6^A modifiers due to limited technology. Hence, comprehensively discerning the TME traits modulated by different m^6^A modifiers may enhance the cognition of antitumor immunity.

Herein, we presented an overall evaluation concerning the interactions of m^6^A modification with TME traits through integration of the HCC transcriptomic and genomic profiles from public data sets. We established three m^6^A modification patterns with diverse outcomes and TME. Also, an m^6^A scoring system was proposed for quantifying the m^6^A modification patterns, which could predict survival outcomes and immunotherapy responses. Thus, m^6^A machinery exerts a nonnegligible function in shaping diverse TME and regulating cancer immunity in HCC.

## 2. Materials and Methods

### 2.1. Acquisition of HCC Cohorts and Preprocessing

Transcriptome profiling and clinicopathological annotation of HCC were gathered from The Cancer Genome Atlas (TCGA) together with Gene-Expression Omnibus (GEO) repositories. Specimens with incomplete follow-up data were removed. For TCGA data set, RNA-seq profiling (FPKM value) of 373 HCC samples and 50 normal samples was gained from the Genomic Data Commons (GDC, https://portal.gdc.cancer.gov/) with TCGAbiolinks package [22]. Afterwards, FPKM was converted to TPM form. Somatic mutations and copy number variations (CNVs) were also retrieved from TCGA. For microarray data from the GSE14520 data set, the raw “CEL” file of 242 HCC samples was retrieved, which was corrected by background and normalized by quantile with robust multiarray averaging algorithm [23]. Through Rcircos package [24], the locations of 23 m^6^A modifiers in chromosome were drawn. The clinical information of TCGA and GSE14520 data sets was shown in Supplementary Tables 1 and 2.

### 2.2. Unsupervised Clustering for 23 m^6^A Regulators

Totally, this study extracted 23 m^6^A modifiers comprising 8 writers (CBLL1, KIAA1429, METTL3/14, RBM15/15B, WTAP, and ZC3H13), 2 erasers (ALKBH5 and FTO), and 13 readers (ELAVL1, FMR1, HNRNPA2B1, HNRNPC, IGF2BP/2/3, LRPPRC, YTHDC1/2, and YTHDF1/2/3) from the obtained data sets. Distinct m^6^A regulator-mediated modification patterns were classified for HCC through unsupervised clustering analyses in the light of the level of aforementioned modifiers. By employing consensus clustering approach, the number and consistency of clustering were determined via ConsensuClusterPlus package with 1000 times repetitions [25].

### 2.3. Clinical Specimens

Three fresh HCC and matched adjacent normal liver tissues were harvested in the Affiliated Traditional Chinese Medicine Hospital of Southwest Medical University from February 2021 to May 2021. No patients experienced preoperative chemo- or radiotherapy before operation. Each subject provided written informed consent following the guideline of the Declaration of Helsinki. The study was approved by the Ethics Committee of The Affiliated Traditional Chinese Medicine Hospital of Southwest Medical University (approval id: 2021017).

### 2.4. Western Blot

Tissue and cell specimens were lysed by RIPA lysis reagent (Beyotime, China) on the ice for half hour and sonicated in an ice bath for 3 min. Following centrifugation at 4°C at 12,000 r/min for 10 min, the supernatant was harvested. The protein concentrations were calculated with BCA kit (Beyotime, China). Then, lysate was boiled with 5 × SDS loading buffer at 100°C for 5 min. Then, protein was separated by SDS-PAGE electrophoresis as well as transferred onto PVDF membranes (Millipore, Germany). Following being washed with TBST, the membranes were blocked by 5% milk/TBST lasting 1 h. Then, the membranes were probed with primary antibodies against METTL3 (1 : 1000; 15073-1-AP; Proteintech, Wuhan, China), ZC3H13 (1 : 1000; DF4623; AFFINITY, USA), YTHDF2 (1 : 1000; 24744-1-AP; Proteintech, Wuhan, China), or GAPDH (1 : 5000; ATA00013Rb; AtaGenix, Wuhan, China) at 4°C overnight. The membranes were washed by PBST for three times. Afterwards, the membranes were exposed to HRP-labeled goat antirabbit secondary antibody (SA00001-2; Proteintech, Wuhan, China) at room temperature for 1 h. The membranes were developed with luminescent buffer and investigated by ChemiDoc™ XRS + gel imaging system (Bio-Rad, Shanghai, China).

### 2.5. Immunofluorescence

Immunofluorescence was performed for detecting METTL3, ZC3H13, and YTHDF2 expression in HCC and normal tissues. In brief, paraformaldehyde-fixed and paraffin-embedded tissue slices were cut into 5 *μ*m thickness. The slices were incubated by anti-METTL3 (1 : 50; 15073-1-AP; Proteintech, Wuhan, China), anti-ZC3H13 (1 : 50; DF4623; AFFINITY, USA), and anti-YTHDF2 (1 : 50; 24744-1-AP; Proteintech, Wuhan, China) antibodies lasting 2 h. Following being washed, the slices were probed with ALexa Fluor 488-conjugated Affinipure goat antirabbit IgG (*H* + *L*) (SA00006-2; Proteintech, Wuhan, China) and DAPI (D9542; Sigma, USA). Then, the sections were mounted with glycerol and investigated under a fluorescence microscope.

### 2.6. Functional Annotation Analyses

The activity of biological processes and pathways between different clusters was compared through gene set variation analysis (GSVA) [26] that represents a nonparametric and unsupervised gene set enrichment method. The Hallmark gene set was retrieved from the Molecular Signatures Database as a reference. Functional annotation analyses of m^6^A regulators or m^6^A-related genes were carried out through clusterProfiler package [27].

### 2.7. Cell Culture and Transfection

HCC cell lines (Hep3B, HUH-7; Chinese Academy of Sciences; Shanghai, China) were grown in DMEM (Thermo Fisher Scientific, USA) plus 10% FBS (Thermo Fisher Scientific, USA), 100 units/mL ampicillin together with 100 *μ*g/mL streptomycin at 37°C with 5% CO_2_. ZC3H13 plasmid was gained from GenePharma company (USA), which was transfected into Hep3B and HUH-7 cells via Lipofectamine™ 2000 transfection reagent (Thermo Fisher Scientific, USA).

### 2.8. 5-Ethynyl-2′-deoxyuridine (EdU) Assay

Cellular proliferation was determined utilizing BeyoClick™ EdU-594 cell proliferation detection kit (C0078S; Beyotime, Shanghai, China). The transfected cells were inoculated onto 24-well plates (8 × 10^5^ cells/well). All operations were carried out following the instructions. Under a fluorescence microscopy, the images were acquired and analyzed.

### 2.9. Transwell Assay

Migration and invasion were examined utilizing Transwell chambers (Corning, Shanghai, China). For invasion test, the chambers were coated by Matrigel (BD, USA), without Matrigel for migration test. HCC cells were inoculated onto the upper chambers plus serum-free media (3 × 10^4^ cells/well). DMEM media with 10% FBS were added to the lower chambers. Following 24 h, migrated or invasive cells were fixed by 4% PFA (Beyotime, China), and dyed utilizing crystal violet. The number of migrated or invaded cells was counted at ×100 magnification utilizing an inverted light microscope.

### 2.10. Assessment of the TME

Single-sample gene set enrichment analyses (ssGSEA) were adopted to infer the relative infiltrations of 28 immune cell populations in the TME. The enrichment scores ranging from 0 to 1 were used to denote the relative infiltrations of each immune population based on the markers of each immune population [28, 29].

### 2.11. Quantifying Immune Response Predictive Factors

The Estimation of Stromal and Immune Cells in Malignant Tumors using Expression Data (ESTIMATE) method was adopted for computing immune/stromal score that could be predictive immune/stromal cell abundance [30]. The Tumor Immune Dysfunction and Exclusion (TIDE) score may infer cancer immunotherapy response [31]. This score was based on two major tumor immune evasion mechanisms: dysfunctional tumor-infiltrating cytotoxic T lymphocytes (CTLs) as well as CTL exclusion via immunosuppressors. Immunophenoscore (IPS) that was developed by four types of immune-related genes: MHC, checkpoint or immunomodulator, effector/suppressor cell population was used to estimate anti-CTLA-4/PD-1 therapeutic response [29].

### 2.12. Dimension Reduction and Ferroptosis Score

Differentially expressed genes (DEGs) with adjusted *p* < 0.05 and |fold-change| > 1.5 were screened between m^6^A methylation patterns with limma package, called as m^6^A [32]. The overlapped DEGs between distinct m^6^A methylation patterns were chosen, called as m^6^A phenotype-associated DEGs. Univariate Cox regression analysis was conducted for screening prognostic m^6^A phenotype-associated DEGs with *p* < 0.05. According to the expression profiling of prognostic m^6^A phenotype-relevant DEGs, HCC subjects were clustered into distinct m^6^A genomic phenotypes. The expression profiles of the prognostic m^6^A phenotype-associated DEGs were utilized for performing PCA, followed by extraction of principal components 1 and 2 as m^6^A score. The approach mostly depended upon the scores on the gene sets with the most favorable association (or inverse association) genes blocks, and down-weighted contribution of genes that cannot be tracked with other set members. The formula [33, 34] was adopted for defining the m^6^A score: m^6^A score = ∑PC1i  +  ∑PC1i, in which *i* denoted m^6^A phenotype-associated DEG level.

### 2.13. Prediction of Chemotherapy and Immunotherapy Response

The response to two commonly chemotherapeutic agents (gemcitabine and cisplatin) was inferred through the Genomics of Drug Sensitivity in Cancer (GDSC; https://www.cancerrxgene.org/) [35]. The half maximal inhibitory concentration (IC50) values were determined through pRRophetic package [36]. The available data of immunotherapy were obtained from IMvigor210 data set in the light of Creative Commons 3.0 License [37]. The immunotherapeutic efficacy was estimated based on subclass mapping (SubMap) analyses [38].

### 2.14. Statistical Analysis

All the computational and statistical analyses were carried out with R programming and GraphPad Prism. Pearson test was applied for assessing the interactions between variables. T-distributed stochastic neighbor embedding (t-SNE) was implemented for the differences between HCC and controls in the light of the mRNA levels of m^6^A modifiers. Kaplan–Meier curves of overall survival (OS), disease-free survival (DFS), disease-specific survival (DSS) together with progression-free interval (PFI) were constructed and the survival differences were computed utilizing log-rank tests. Univariate and multivariate analyses were utilized for assessing the independency of variables in predicting prognosis. Comparisons between two groups were presented with student's *t* or Wilcoxon tests, with one-way analysis of variance or Kruskal–Wallis test among more than two groups. *p* values < 0.05 were regarded as statistical significance.

## 3. Results

### 3.1. Gene Mutations and Expression of m^6^A Regulators in HCC

**Figure 1:**
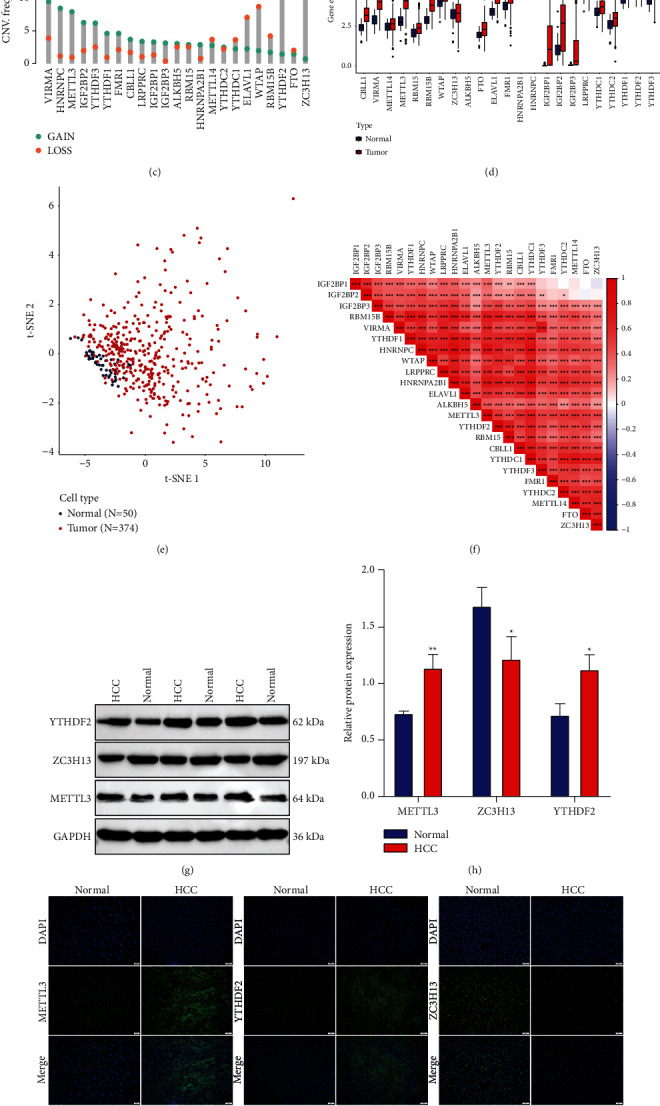
Overall mutations and expression of m^6^A regulatory genes across HCC: (a) the locations of CNV alterations of 23 m^6^A modifiers on human chromosome. (b) The frequency of somatic mutations of 23 m^6^A modifiers across HCC specimens from TCGA cohort. The number on the right denoted the variation frequencies of modifies and the right bars displayed the proportions of mutation types. Each column represented individual samples. The stacked bars below indicated the fractions of transformation across specimens. (c) The CNV frequencies of m^6^A modifiers across HCC specimens from TCGA cohort. The column height indicated the mutation frequencies. Blue represented the frequency of gain and orange represented the frequency of loss. (d) The differences in mRNA levels of 23 m^6^A modifiers between HCC and normal tissues in TCGA cohort. (e) The t-SNE for distinguishing HCC (red dot) from control (blue dot) specimens based on the regulators. (f) Pearson analyses for the mutual regulation among regulators at the mRNA expression. The darker the red, the stronger the correlation. (g, h) Western blot of the expression of three regulators METTL3, ZC3H13, YTHDF2 in 3 paired HCC and normal tissues. (i, j) Immunofluorescence for the levels of above regulators. Bar = 50 *μ*m. Ns: no significance; ^*∗*^*p* < 005; ^*∗∗*^*p* < 0.01; ^*∗∗∗*^*p* < 0.001; ^*∗∗∗∗*^*p* < 0.0001.

Here, the present research observed the roles of 23 m^6^A modifiers across HCC comprising 8 writers, 2 erasers, and 13 readers (Figure 1(a)). The prevalence of somatic variations of above regulators in HCC was summarized in Figure 1(b). Totally, 39 of the 364 (10.71%) HCC specimens displayed somatic variations of m^6^A regulators, mainly containing missense mutation, nonsense mutation, splice site, in frame deletion, and frame shift deletion. HNRNPC, LRPPRC, ZC3H13, ELAVL1, FMR1, RNPA2B1, YTHDC1, YTHDC2, WTAP, and IGF2BP3 occurred somatic variations in HCC. Further analysis revealed the prevalent CNVs in m^6^A regulators and gain was the most frequent CNV type (Figure 1(c)). We further ascertained whether the aforementioned gene mutations affected the mRNA level of m^6^A modifiers across HCC. Compared to control specimens, most exhibited higher expression in HCC specimens (Figure 1(d)). The m^6^A regulators with gain CNVs significantly had increased expression in HCC. This indicated that CNVs might prominently contribute to the perturbation on the m^6^A modifier level. In accordance with 23 m^6^A modifiers, HCC specimens were distinctly distinguished from control specimens (Figure 1(e)). Pearson correlation analyses indicated the significantly mutual regulation between regulators, as shown in Figure 1(f). Among 23 m^6^A regulators, we selected three regulators METTL3, ZC3H13, and YTHDF2 to verify their expression in three paired HCC and normal tissues. Our Western blot (Figures 1(g) and 1(h)) and immunofluorescence assays (Figures 1(i) and 1(j)) confirmed that METTL3 and YTHDF2 were significantly up-regulated, while ZC3H13 possessed distinct down-regulation in HCC *versus* normal specimens. These data unveiled the heterogeneity in mutations and levels of m^6^A modifiers between HCC and controls together with their important implications in liver tumorigenesis.

### 3.2. Prognostic Implications and Biological Functions of m^6^A Regulators in HCC

**Figure 2:**
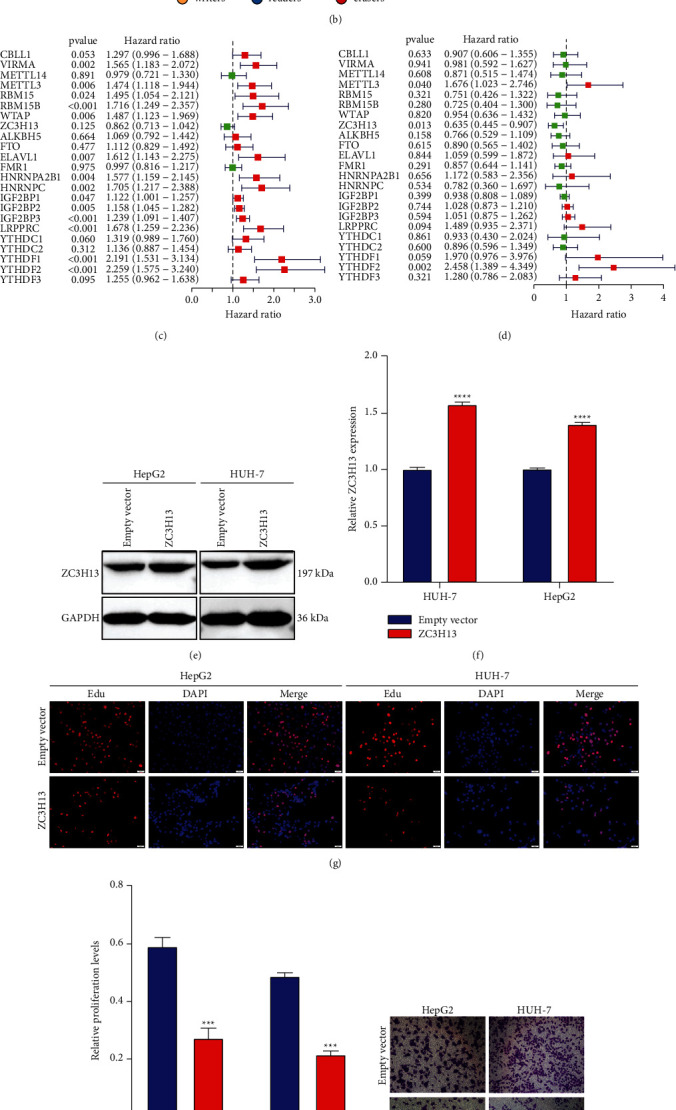
Characterization of prognostic implications and biological functions of 23 m^6^A regulators across HCC: (a) functional annotations of the regulators with GO enrichment analyses. The bar length represented the number of enriched regulators. (b) The interaction of expression on 23 m^6^A regulators (writers, yellow; readers, blue; erasers, red) in HCC. The lines that connected modifiers indicated their interactions, and the circle size indicated the survival value of regulators and scaled by *p* values. (c) Uni- and (d) multivariate cox regression analyses of 23 m^6^A regulators in HCC prognosis. (e, f) Western blot of the expression of ZC3H13 in HeG2 and HUH-7 cells transfected with empty vector and ZC3H13 up-regulation. (g, h) EdU staining for cell proliferation of HeG2 and HUH-7 cells with empty vector and ZC3H13 up-regulation. (i–l) Transwell assays for the migration and invasion of HeG2 and HUH-7 cell lines following transfection with empty vector and ZC3H13 overexpression. (m) Heatmap showing the top 30 genes associated with ZC3H13 in HCC. ^*∗∗∗*^*p* < 0.001; ^*∗∗∗∗*^*p* < 0.0001.

Functional annotation analyses confirmed the influence of 23 m^6^A regulators on mRNA methylation, RNA modification, RNA stability, and the like (Figure 2(a)). As depicted in Figure 2(b), there were close correlations between 23 writer, reader, and eraser modifiers. This indicated the functions of distinct m^6^A modifiers on HCC pathogenesis. Univariate and multivariate analyses showed the survival implication of above regulators (Figures 2(c) and 2(d)). Among them, METTL3, ZC3H13, and YTHDF2 could act as independent prognostic indicators. Previous research has reported that METTL3 and YTHDF2 may facilitate HCC progression [39]. Here, we observed the biological functions of ZC3H13 in HCC. In HepG2 and HUH-7 cells, ZC3H13 was successfully overexpressed following transfection with ZC3H13 plasmid (Figures 2(e) and 2(f)). EdU staining showed that ZC3H13 overexpression markedly weakened proliferation of HepG2 and HUH-7 cells (Figures 2(g) and 2(h)). Also, the migrated (Figures 2(i) and 2(j)) and invasive capacities (Figures 2(k) and 2(l)) were distinctly suppressed in HCC cells after overexpressing ZC3H13. Figure 2(m) showed the top-30 genes associated with ZC3H13 in HCC. Collectively, m^6^A regulators possessed the important prognostic implications and biological functions in HCC.

### 3.3. Characterization of m^6^A Regulator Expression Patterns with Distinct Prognosis and TME Landscape

**Figure 3:**
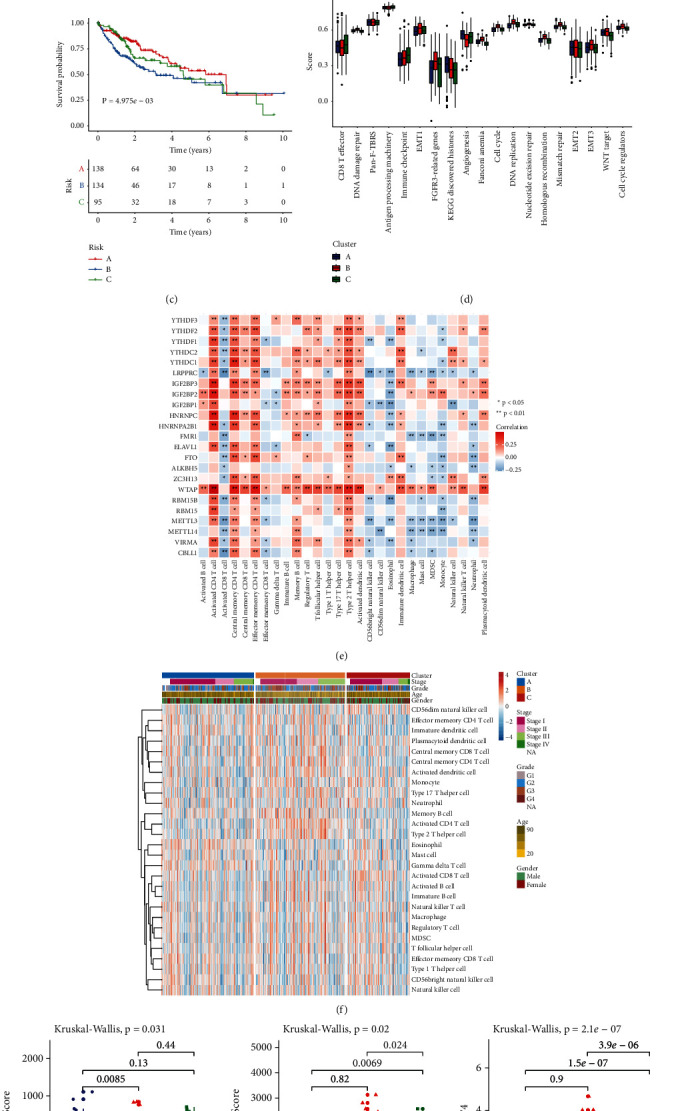
Characterization of m^6^A methylation patterns with diverse prognosis and TME landscape in TCGA cohort: (a) Heatmaps for the mRNA levels of 23 m^6^A modifiers among three m^6^A machinery phenotypes. (b) Heatmap of the GSVA scores of Hallmark pathways in distinct m^6^A modification patterns. (c) Kaplan–Meier OS curves for HCC patients from TCGA data set with diverse patterns (log-rank tests). (d) The differences in the activation of several pathways among three patterns. Kruskal-Wallis H test was applied for comparing three clusters. Ns: no significance; ^*∗*^*p* < 0.05; ^*∗∗*^*p* < 0.01; ^*∗∗∗*^*p* < 0.001. (e) Heatmap of the correlations of 23 m^6^A modifiers with infiltrating immune cells based on ssGSEA method. Positive correlation, red; negative correlation, blue. (f) Heatmap for the differences in 28 immune population abundance among three patterns. (g) The discrepancy in stromal/immune score and CD274 expression among three patterns.

Our aforementioned data indicated that the interplay between m^6^A regulators exerted a key function in taking shape diverse m^6^A modification patterns of HCC. Through ConsensusClusterPlus package, we classified HCC subjects in TCGA cohort as diverse m^6^A modification patterns in the light of the mRNA levels of 23 m^6^A regulatory genes. As a result, three distinct modification patterns were characterized with unsupervised clustering analyses (Supplementary Figures 1(a)–1(d)). Our data showed the distinct discrepancy in m^6^A regulator levels among three m^6^A modification patterns (Figure 3(a)). For most m^6^A regulators, cluster B displayed the highest expression, with the moderate expression in cluster A and the lowest expression in cluster C. For exploring the biological molecular alterations underlying three clusters, GSVA was carried out based on the Hallmark gene set (Figure 3(b)). We found that cluster A was markedly enriched in metabolism-related processes (fatty acid/bile acid/xenobiotic metabolisms, etc.). Meanwhile, cluster B displayed prominently enriched pathways that were in relation to carcinogenic activation, stromal and immune pathways like PI3K-Akt-mTOR, P53, Hedgehog, Notch, Wnt-*β*-catenin, TGF-*β*, complement, IL2-STAT5, and IL6-JAK-STAT3 pathways. Nevertheless, most pathways were down-regulated in cluster C. Prognosis analysis showed that cluster A presented a distinct survival superiority, while cluster B possessed the poorest outcomes in TCGA data set (Figure 3(c)). Further analyses confirmed that stromal activation was distinctly strengthened in cluster B, as displayed by EMT and WNT-target processes (Figure 3(d)). Genetic mutation pathways were also markedly activated in cluster B including DNA damage repair, DNA replication, nucleotide excision repair, homologous recombination, and mismatch repair, with cell cycle-related processes in cluster B. The ssGSEA approaches were adopted for estimating the immune population abundance across HCC. Pearson correlation analysis indicated the significantly positive or negative interactions between 23 m^6^A modifiers and infiltrating immune populations (Figure 3(e)). Heatmap was depicted for visualizing the differences in immune population abundance among patterns. Antitumor lymphocyte populations like effector memory CD4+ T, activated CD4+ T, and natural killer cells exhibited primary activation in cluster B (Figure 3(f)). By ESTIMATE approach, this study quantified the overall infiltrations of immune together with stromal cell populations. Cluster A was characterized by enhanced stromal score, cluster C displayed relatively high immune score and cluster B had the highest CD274 (PD-L1) expression (Figure 3(g)).

### 3.4. Construction of m^6^A Genomic Phenotypes in HCC

**Figure 4:**
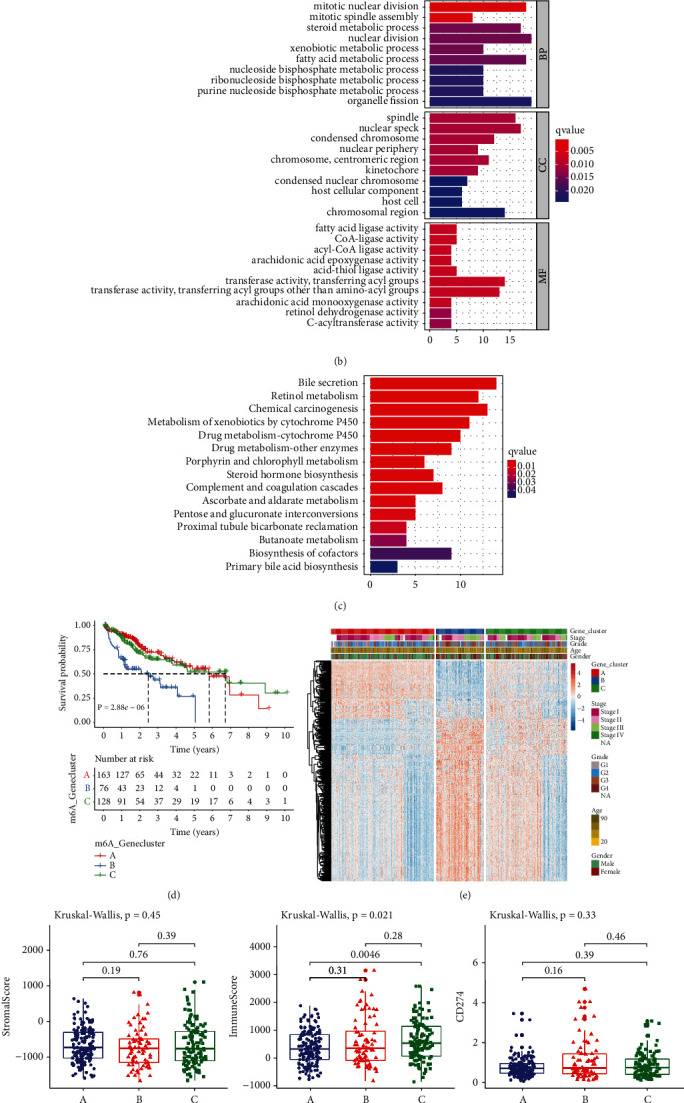
Construction of m^6^A genomic phenotypes in HCC from TCGA cohort: (a) Venn diagram for the 331 m^6^A phenotype-associated DEGs between three genomic phenotypes. (b) GO and (c) KEGG enrichment results of the m^6^A phenotype-associated DEGs. (d) Kaplan–Meier curves of OS for three gene clusters (log-rank tests). (e) Heatmap for the levels of the m^6^A phenotype-associated DEGs among clusters. (f) The discrepancy in stromal score, immune score and CD274 expression among clusters.

For more deeply investigating the underlying biological behaviors in each m^6^A cluster, 331 overlapping genes with deregulation were selected among distinct m^6^A modification patterns with limma approach, called as m^6^A phenotype-associated DEGs (Figure 4(a); Supplementary Table 3). Functional annotation analyses showed the distinct enrichment of the m^6^A phenotype-associated DEGs in metabolic processes (Figure 4(b)) and pathways (Figure 4(c)), confirming the critical roles of m^6^A methylation in HCC progression. For validating the regulation mechanisms, unsupervised clustering analysis was carried out according to above 331 m^6^A phenotype-associated DEGs. Consistent with the m^6^A modification patterns, patients were classified as three genomic phenotypes (Supplementary Figures 2(a)–2(d)). Prognosis analysis showed that gene cluster B displayed the worst survival outcomes among three genomic phenotypes (Figure 4(d)). Heatmap visualized the expression of the m^6^A phenotype-associated DEGs among three gene clusters (Figure 4(e)). Consistent with m^6^A modification patterns, gene cluster B displayed the largest stromal score, with the highest immune score in cluster C and the activated CD274 expression in cluster B (Figure 4(f)).

### 3.5. Establishment of an m^6^A Scoring System and Characterization of Its Clinical Implications

**Figure 5:**
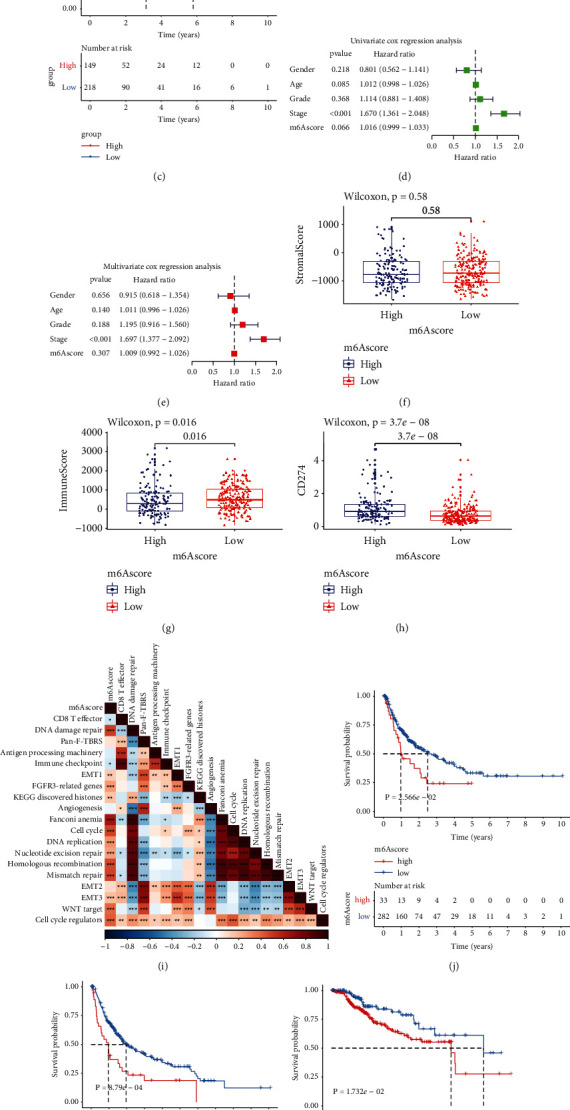
Establishment of an m^6^A scoring system along with characterization of its clinical implications in TCGA cohort: (a) alluvial chart of m^6^A modification, gene cluster, m^6^A score along with survival status. (b) The discrepancy in m^6^A score between three m^6^A-modified gene clusters. *P* values were calculated with Kruskal-Wallis tests. (c) Kaplan–Meier OS curves for patients high or low m^6^A score (log-rank test). (d) Uni- and (e) multivariate analyses of the independency of the m^6^A score in predicting HCC outcomes. (f–h) The discrepancy in (f) stromal score, (g) immune score and (h) CD274 expression between groups. *P* values were determined using Wilcoxon tests. (i) Associations between m^6^A score and several pathways in HCC specimens. (j–m) Kaplan–Meier curves of (j) DFI, (k) DFS, (l) DSS and (m) PFI for cases with high or low m^6^A scores (log-rank tests). ^*∗*^*p* < 005; ^*∗∗*^*p* < 0.01; ^*∗∗∗*^*p* < 0.001; ^*∗∗∗∗*^*p* < 0.0001.

For accurately predicting m^6^A machinery classification across each HCC sample, this study generated the m^6^A scoring system according to the prognostic m^6^A phenotype-associated DEGs (Figure 5(a)). We compared m^6^A score among distinct m^6^A genomic phenotypes. Consequently, genomic cluster B exhibited the largest m^6^A score, whereas cluster A possessed the lowest m^6^A score (Figure 5(b)). Prognosis analysis showed that high m^6^A score indicated poorer OS than low m^6^A score (Figure 5(c)). Following univariate and multivariate analyses, m^6^A score acted as an independent survival indicator (Figures 5(d) and 5(e)). After stratifying HCC cases into diverse subgroups in the light of clinicopathological traits (age, sex, grade, and stage), patients with high m^6^A score also displayed unfavorable outcomes than those with low m^6^A score (Supplementary Figure 3(a)–3(h)). No notable differences in stromal score were measured between high and low m^6^A scores (Figure 5(f)). Nevertheless, low m^6^A score displayed the increased immune score compared to high m^6^A score (Figure 5(g)). CD274 expression was also compared between groups. In Figure 5(h), high m^6^A score was characterized by elevated CD274 expression. We also found that m^6^A score was positively associated with stromal pathways (including EMT and WNT-target) and genetic mutation-related pathways (Figure 5(i)). Furthermore, high m^6^A score indicated the poorer DFI (Figure 5(j)), DFS (Figure 5(k)), DSS (Figure 5(l)), and PFI (Figure 5(m)) compared to low m^6^A score, indicating that m^6^A score could be utilized for predicting HCC recurrence and progression.

### 3.6. The m^6^A Score Can Predict Chemotherapeutic and Immunotherapeutic Benefits

**Figure 6:**
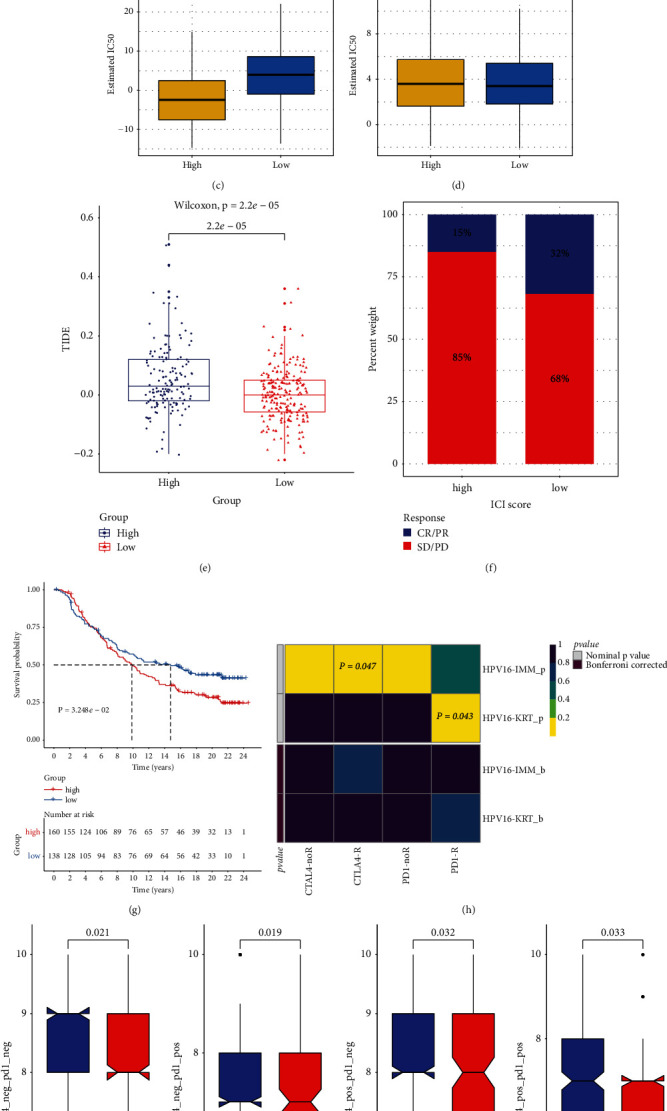
The roles of m^6^A score in prediction of chemo- and immunotherapeutic benefits of HCC patients in TCGA cohort: (a, b) Somatic mutation landscape of significantly mutated genes across (a) high or (b) low m^6^A score populations. (c, d) The differences in estimated IC50 values between populations. (e) The comparisons in TIDE score between populations. *P* values were determined with Wilcoxon tests. (f) The proportions of subjects responding to anti-PD-L1therapy in two populations in the IMvigor210 cohort. (g) Kaplan–Meier OS curves for cases with high and low m^6^A scores (log-rank test). (h) The response of cases with high and low m^6^A scores to anti-CTLA-4/PD-1 therapies through SubMap analyses. (i) The comparisons of IPS scores in two populations.

Tumor somatic mutations were compared between m^6^A score subgroups in TCGA cohort. As depicted in Figures 6(a) and 6(b), more prevalent somatic mutations occurred in high m^6^A group compared to low m^6^A group. The responses to two commonly chemotherapeutic drugs were assessed by GDSC database. After comparison, the estimated IC50 value of gemcitabine was markedly lower in high m^6^A score population (Figure 6(c)). This demonstrated that patients possessing high m^6^A score were more likely to benefit from gemcitabine. Nevertheless, no significant difference in cisplatin was found between groups (Figure 6(d)). TIDE score, an emerging predictor of immunotherapy, was calculated in each HCC specimen. Compared to high m^6^A score group, decreased TIDE score was found in low m^6^A score population (Figure 6(e)), indicating the more benefits from immunotherapy. Figure 6(f) visualized the proportions of patients that responded to anti-PD-L1 immunotherapy in the IMvigor210 data set. The responser/nonresponser was 15%/85% in high m^6^A score population and 32%/68% in another population. This confirmed that cases with low m^6^A score exhibited the distinct therapeutic advantage in anti-PD-L1 therapy. In addition, we compared the survival differences between populations in this cohort. In Figure 6(g), cases with low m^6^A score possessed the significant prognostic advantage. SubMap analyses also indicated that low m^6^A score cases possessed the greater possibility of responding to anti-PD-1 therapy (Figure 6(h)). The IPS score was determined for evaluating the immune response, which was markedly increased in low m^6^A score population (Figure 6(i)). Thus, m^6^A modification patterns could be involved in mediating the tumor immune response.

### 3.7. External Validation of m^6^A Methylation Patterns and m^6^A Score

**Figure 7:**
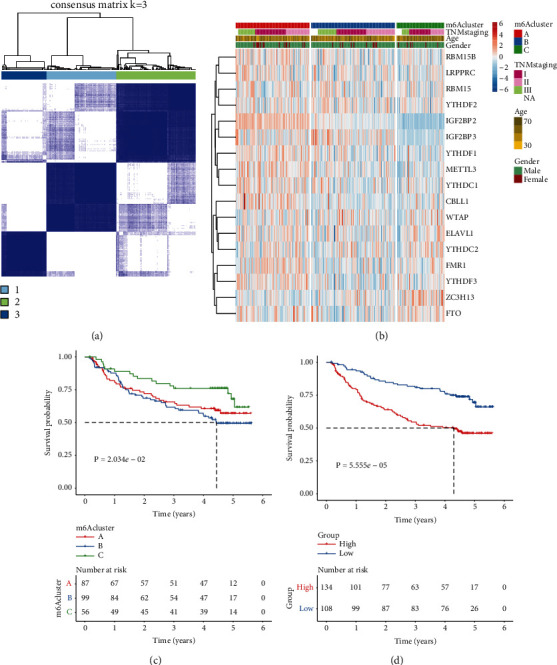
Verification of the m^6^A methylation patterns and m^6^A score for HCC in the GSE14520 data set: (a) Consensus clustering analysis for classifying 242 HCC patients into three m^6^A machinery phenotypes in the light of the expression profiles of 23 m^6^A modifiers. (b) Heatmap for the expression of 23 m^6^A regulators among three patterns. (c) Kaplan–Meier OS curves among three phenotypes. (d) Kaplan-Meier OS curves for cases with high or low m^6^A score. *P* values were calculated with log-rank tests.

The m^6^A regulator-based m^6^A methylation patterns were validated among 242 HCC patients in the GSE14520 cohort. Consistent with the TCGA cohort, HCC cases were classified as three m^6^A methylation patterns (Figure 7(a)). Heatmap visualized the notable discrepancy in 23 m^6^A modifier levels among three m^6^A methylation patterns (Figure 7(b)). Similarly, cluster B exhibited the poorest OS outcomes (Figure 7(c)). Based on the m^6^A-related DEGs, we also calculated m^6^A score of each HCC case in the GSE14520 data set. Data demonstrated that cases with low m^6^A score exhibited the significant survival advantage (Figure 7(d)). These data confirmed the accurate classifications of HCC based on 23 m^6^A regulators.

## 4. Discussion

Mounting evidences suggest that m^6^A machinery is closely in relation to innate immunity, inflammatory response along with anticancer effects by interaction with different m^6^A regulatory genes [40]. Here, we established three m^6^A regulator-based m^6^A modification phenotypes with diverse prognostic outcomes, biological processes along with TME traits across HCC according to the expression profiling of 8 writers, 2 erasers, and 13 readers. Based on m^6^A-associated DEGs, we developed an m^6^A scoring system for each HCC specimen. The m^6^A score enabled to infer immunotherapeutic response. Hence, the current research highlighted the roles of m^6^A machinery in shaping TME along with immunity modulation, which might promote precision immunotherapeutic strategies.

Herein, we comprehensively uncovered the somatic mutations, CNVs and expression patterns of 23 m^6^A regulators. Most displayed the gain CNVs and high expression in HCC, and their expression profiling could distinguish HCC from normal liver tissues. Also, most were distinctly correlated to HCC prognosis. These data suggested that m^6^A regulators participated in HCC initiation and progress. We verified METTL3, ZC3H13, and YTHDF2 levels across HCC and normal specimens via Western blot and immunofluorescence. Our data confirmed the up-regulation of METTL3 and YTHDF2 as well as the down-regulation of ZC3H13 in HCC. So far, there is no study about the implications of ZC3H13 across HCC. However, previous research has found that ZC3H13 acts as a tumor suppressor gene in breast carcinoma [41]. It mitigates growth along with invasive capacity of colorectal cancer via inactivation of Ras-ERK signaling [42]. Herein, our experiments showed that ZC3H13 up-regulation exerted an inhibitory effect on proliferation and invasion of HCC cells, confirming the anti-HCC effects of ZC3H13.

At the mRNA levels, 23 m^6^A regulators exhibited significant synergistic effects in HCC. Hence, the current research established three m^6^A machinery patterns in the light of their expression. In cluster A, metabolism-related processes were distinctly activated while cluster B displayed the activation of carcinogenic, stromal, and immune pathways. This explained why cluster B had the worst survival outcomes. The three m^6^A methylation patterns were characterized by distinct TME. For instance, anti-tumor lymphocyte cells were mainly activated in cluster B. Totally, 331 DEGs were identified among patterns, which were mainly in relation to metabolic processes. The evidence suggests the biological implications of metabolic process in HCC outcomes [2]. On the basis of m^6^A-associated DEGs, three m^6^A gene phenotypes were clustered. Consistent with the m^6^A methylation patterns, three gene phenotypes were characterized by distinct prognosis and TME. For defining m^6^A modification patterns, we developed an m^6^A scoring system, which might guide treatment plans for each subject. Our prognosis analyses unveiled that the m^6^A score acted as a credible independent survival predictor for HCC. Elevated m^6^A score was markedly correlated to undesirable OS, recurrence, and progression of HCC patients. Moreover, m^6^A score exhibited the strong correlations to immune predictors such as TIDE and IPS. Low m^6^A score was more likely to be responsive to anti-PD-1/PD-L1 therapy in the IMvigor210 cohort. Our data confirmed that the m^6^A machinery patterns can be adopted in clinical application as well as immunotherapy plans for HCC.

There are advantages in our study. First, several experimental studies have verified the roles of single m^6^A regulators in HCC [43, 44]. For instance, RBM15 is highly expressed in HCC, and its up-regulation is indicative of undesirable survival outcomes as well as triggers HCC progression by modulating m^6^A-modified YES1 depending upon IGF2BP1 [45]. However, the function of ZC3H13 in HCC progression remains unclear. Herein, this research for the first time confirmed the low level of ZC3H13 in HCC tissue and ZC3H13 up-regulation inhibited the proliferation, migration along with invasion of HCC cells through experiments. Second, although a recent study constructed distinct m^6^A regulator-based m^6^A modification patterns for HCC in TCGA cohort, this classification was not externally verified in independent cohorts. Herein, we determined three m^6^A regulator-mediated modification patterns for HCC, and confirmed the classification accuracy in the GSE14520 cohort. Third, we developed an m^6^A score that could predict survival outcomes and therapeutic responses for HCC patients. Despite this, several limitations should be pointed out. First, although the current research reviewed the literature and collected 23 known m^6^A modifiers, new identified regulators should be included for optimizing the accuracy of the m^6^A machinery phenotypes. Second, the m^6^A machinery phenotypes and m^6^A score were proposed based on retrospective cohorts. Therefore, more prospective cohorts of HCC cases are needed to verify our conclusion.

## 5. Conclusion

Collectively, the current research synthetically evaluated the m^6^A regulator-based m^6^A machinery phenotypes for HCC that were characterized by different prognosis, TME features, and activation of biological processes. Moreover, quantification of the m^6^A modification patterns by m^6^A score may enhance the cognition of TME features as well as offer key insights into immunotherapy responses.

## Data Availability

The data used to support the findings of this study are included within the supplementary materials.

## Abbreviations

HCC: Hepatocellular carcinoma
m^6^A: N^6^-methyladenosine
mRNA: Messenger RNA
TME: Tumor microenvironment
TCGA: The cancer genome atlas
GEO: Gene-expression omnibus
CNV: Copy number variation
GSVA: Gene set variation analysis
EdU: 5-Ethynyl-2′-deoxyuridine
ssGSEA: Single sample gene set enrichment analyses
TIDE: Tumor immune dysfunction and exclusion
DEGs: Differentially expressed genes
GDSC: Genomics of drug sensitivity in cancer
IC50: Half maximal inhibitory concentration
t-SNE: T-distributed stochastic neighbor embedding
OS: Overall survival
DFS: Disease-free survival
DSS: Disease-specific survival
PFI: Progression-free interval.

## Supplementary Materials

Supplementary Materials Supplementary Figure 1. Consensus clustering analyses of stratifying HCC cases in TCGA cohort into three m^6^A methylation patterns according to 23 m^6^A regulators. (A) Heatmap for the consensus matrix *k* = 3. (B) Cumulative distribution function (CDF) under diverse *k* values. (C) Delta area diagram for relative alterations in area under CDF curves. (D) The tracking plot for HCC samples under different *k* values. Supplementary Figure 2. Consensus clustering analyses for clustering three m^6^A genomic phenotypes in the light of the expression profiling of m^6^A-associated genes in TCGA cohort. (A) Heatmap for the consensus matrix *k* = 3. (B) CDF under diverse *k* values. (C) Delta area diagram for relative alterations in area under CDF curves. (D) The tracking plot for HCC samples under different *k* values. Supplementary Figure 3. Subgroup analysis of the prognosis value of m^6^A score among HCC patients in TCGA data set. Kaplan-Meir curves of cases with high or low m^6^A score in each subgroup: (A) age ≥ 65; (B) age < 65; (C) female; (D) male; (E) G1-2; (F) G3-4; (G) stage I-II; (H) stage III-IV. *P* values were determined through log-rank tests. Supplementary Table 1. The clinical information of HCC samples in the TCGA data set. Supplementary Table 2. The clinical information of HCC samples in the GSE14520 data set. Supplementary Table 3. The list of 331 m^6^A phenotype-associated DEGs.
