# Supplementary material for: M6A Modifier-Mediated Methylation Characterized by Diverse Prognosis, Tumor Microenvironment, and Immunotherapy Response in Hepatocellular Carcinoma
Source: J Oncol. 2022 Aug 16;2022:2513813. doi: 10.1155/2022/2513813 (PMC9398803; doi:10.1155/2022/2513813)
Supplement: Supplementary Materials — Supplementary Figure 1. Consensus clustering analyses of stratifying HCC cases in TCGA cohort into three m6A methylation patterns according to 23 m6A regulators. (A) Heatmap for the consensus matrix k = 3. (B) Cumulative distribution function (CDF) under diverse k values. (C) Delta area diagram for relative alterations in area under CDF curves. (D) The tracking plot for HCC samples under different k values. Supplementary Figure 2. Consensus clustering analyses for clustering three m6A genomic phenotypes in the light of the expression profiling of m6A-associated genes in TCGA cohort. (A) Heatmap for the consensus matrix k = 3. (B) CDF under diverse k values. (C) Delta area diagram for relative alterations in area under CDF curves. (D) The tracking plot for HCC samples under different k values. Supplementary Figure 3. Subgroup analysis of the prognosis value of m6A score among HCC patients in TCGA data set. Kaplan-Meir curves of cases with high or low m6A score in each subgroup: (A) age ≥ 65; (B) age < 65; (C) female; (D) male; (E) G1-2; (F) G3-4; (G) stage I-II; (H) stage III-IV. P values were determined through log-rank tests. Supplementary Table 1. The clinical information of HCC samples in the TCGA data set. Supplementary Table 2. The clinical information of HCC samples in the GSE14520 data set. Supplementary Table 3. The list of 331 m6A phenotype-associated DEGs. [file 2513813.f1.zip › 2513813.f1/Supplementary table 1.pdf]

Supplementary table 1. The clinical information of HCC samples in the TCGA dataset.

Sample ID	OS	Status	Gender	Age	Grade	Stage	M	N
TCGA-2Y-A9GS-01	23.78	1	Male	58	G2	NA	MX	NX
TCGA-2Y-A9GT-01	53.35	1	Male	51	G2	Stage I	MX	NX
TCGA-2Y-A9GU-01	63.7	0	Female	55	G2	Stage I	MX	NX
TCGA-2Y-A9GV-01	83.18	1	Female	54	G1	Stage I	MX	NX
TCGA-2Y-A9GW-01	41.75	1	Male	64	G2	Stage I	MX	N0
TCGA-2Y-A9GX-01	80.22	0	Male	68	G2	Stage I	MX	NX
TCGA-2Y-A9GY-01	24.87	1	Female	64	G3	Stage II	MX	NX
TCGA-2Y-A9GZ-01	27.86	1	Female	82	G2	Stage II	MX	NX
TCGA-2Y-A9H0-01	120.73	0	Male	49	G1	Stage IIIA	M0	N0
TCGA-2Y-A9H1-01	40.37	1	Male	58	G2	Stage I	MX	NX
TCGA-2Y-A9H2-01	56.87	0	Female	64	G3	Stage I	MX	N0
TCGA-2Y-A9H3-01	49.8	0	Male	45	G1	Stage II	MX	NX
TCGA-2Y-A9H4-01	47.7	0	Male	68	G2	Stage I	MX	N0
TCGA-2Y-A9H5-01	18.23	1	Female	59	G3	Stage I	MX	N0
TCGA-2Y-A9H6-01	11.73	0	Female	68	G2	Stage I	MX	NX
TCGA-2Y-A9H7-01	38.37	0	Female	81	G2	Stage I	MX	N0
TCGA-2Y-A9H8-01	20.8	1	Female	85	G2	NA	MX	NX
TCGA-2Y-A9H9-01	22.9	0	Male	70	G2	Stage I	MX	N0
TCGA-2Y-A9HA-01	1.18	1	Male	70	G2	Stage II	MX	NX
TCGA-2Y-A9HB-01	8.54	0	Male	66	G2	Stage I	MX	NX
TCGA-3K-AAZ8-01	13.01	0	Male	65	G1	Stage IIIB	MX	NX
TCGA-4R-AA8I-01	8.61	1	Male	66	G2	Stage II	MX	NX
TCGA-5C-A9VG-01	10.78	0	Male	58	G2	Stage II	M0	N0
TCGA-5C-A9VH-01	10.58	0	Male	70	G2	Stage I	M0	N0
TCGA-5C-AAPD-01	0.66	0	Male	61	G1	Stage II	M0	N0
TCGA-5R-AA1C-01	17.08	0	Male	57	G2	Stage II	M0	N0
TCGA-5R-AA1D-01	14.75	0	Female	17	G3	Stage IIIA	M0	N0
TCGA-5R-AAAM-01	1.51	1	Female	65	G2	Stage II	M0	N0
TCGA-BC-4072-01	48.95	1	Female	74	G3	Stage IIIA	M0	N0
TCGA-BC-4073-01	27.89	0	Male	73	G3	Stage IIIA	MX	N0
TCGA-BC-A10Q-01	37.29	1	Female	72	NA	NA	MX	NX
TCGA-BC-A10R-01	10.12	1	Female	66	G2	NA	MX	NX
TCGA-BC-A10S-01	46.75	1	Male	81	G1	NA	MX	NX
TCGA-BC-A10T-01	27.5	1	Male	76	G1	NA	MX	NX
TCGA-BC-A10U-01	27.5	1	Male	69	G2	NA	MX	NX
TCGA-BC-A10W-01	2.99	1	Male	50	G3	NA	MX	NX
TCGA-BC-A10X-01	25.3	1	Female	52	G2	Stage IIIA	MX	N0
TCGA-BC-A10Y-01	23.36	1	Male	76	G3	NA	MX	NX
TCGA-BC-A10Z-01	1.12	1	Female	62	G2	Stage I	MX	N0
TCGA-BC-A110-01	69.51	1	Female	51	G1	NA	MX	NX
TCGA-BC-A112-01	5.03	1	Male	80	G2	NA	MX	NX
TCGA-BC-A216-01	44.38	0	Female	62	G2	Stage IIIA	M0	NX
TCGA-BC-A217-01	45.89	1	Female	75	G3	Stage II	M0	NX
TCGA-BC-A3KF-01	0.26	0	Female	66	G2	Stage I	M0	NX
TCGA-BC-A3KG-01	22.34	0	Female	68	G3	Stage II	M0	N0
TCGA-BC-A5W4-01	17.97	1	Male	69	G3	Stage IIIA	M0	NX
TCGA-BC-A69H-01	14.59	0	Male	64	G3	Stage II	M0	NX
TCGA-BC-A69I-01	12.71	0	Male	69	G1	Stage I	M0	N0
TCGA-BC-A8YO-01	18.46	0	Female	66	G3	Stage IIIC	M0	N0
TCGA-BD-A2L6-01	44.78	0	Male	69	G2	NA	MX	NX

TCGA-BD-A3EP-01	13.44	0	Female	75	G2	Stage I	M0	N0
TCGA-BD-A3ER-01	36.63	0	Male	62	G2	Stage II	MX	NX
TCGA-BW-A5NO-01	0.66	0	Male	50	G2	Stage IIIA	MX	NX
TCGA-CC-5258-01	4.24	1	Male	48	G2	Stage II	M0	N0
TCGA-CC-5259-01	8.21	0	Female	60	G2	Stage IIIC	M0	N0
TCGA-CC-5260-01	2.86	1	Female	61	G1	Stage IIIC	M0	N0
TCGA-CC-5261-01	3.19	1	Male	44	G2	Stage II	M0	N0
TCGA-CC-5262-01	3.38	1	Male	67	G1	Stage IIIC	M0	N0
TCGA-CC-5263-01	4.24	1	Male	35	G1	Stage IIIA	M0	N0
TCGA-CC-5264-01	3.35	1	Male	71	G2	Stage IIIA	M0	N0
TCGA-CC-A123-01	7.19	0	Female	24	G1	Stage IIIA	M0	N0
TCGA-CC-A1HT-01	3.32	1	Male	50	G3	Stage IIIA	M0	N0
TCGA-CC-A3M9-01	9.86	1	Male	45	G3	Stage IIIA	M0	N0
TCGA-CC-A3MA-01	9.95	1	Male	61	G2	Stage IIIA	M0	N0
TCGA-CC-A3MB-01	10.35	1	Male	36	G1	Stage IIIA	M0	N0
TCGA-CC-A3MC-01	11.93	0	Male	54	G2	Stage IIIA	M0	N0
TCGA-CC-A5UC-01	11.4	1	Male	63	G3	Stage IIIA	M0	N0
TCGA-CC-A5UD-01	9.99	1	Male	45	G2	Stage IIIA	M0	N0
TCGA-CC-A5UE-01	8.94	1	Male	48	G2	Stage IIIB	M0	N0
TCGA-CC-A7IE-01	7.13	1	Male	57	G2	Stage IIIA	M0	N0
TCGA-CC-A7IF-01	21.32	1	Male	59	G1	Stage IIIA	M0	N0
TCGA-CC-A7IG-01	9.82	1	Male	47	G2	Stage II	M0	N0
TCGA-CC-A7IH-01	11.99	0	Male	58	G1	Stage IIIA	M0	N0
TCGA-CC-A7II-01	13.11	0	Male	54	G3	Stage IIIA	M0	N0
TCGA-CC-A7IJ-01	12.55	0	Male	56	G3	Stage II	M0	N0
TCGA-CC-A7IK-01	8.61	1	Male	59	G3	Stage IIIA	M0	N0
TCGA-CC-A7IL-01	9.13	1	Male	61	G1	Stage IIIA	M0	N0
TCGA-CC-A8HS-01	9.86	1	Male	18	G1	Stage IIIC	M0	N1
TCGA-CC-A8HT-01	4.6	1	Male	74	G2	Stage IIIA	M0	N0
TCGA-CC-A8HU-01	11.3	1	Female	39	G3	Stage IIIA	M0	N0
TCGA-CC-A8HV-01	9.17	1	Female	51	G2	Stage II	M0	N0
TCGA-CC-A9FS-01	6.93	0	Male	55	G2	Stage II	M0	N0
TCGA-CC-A9FW-01	8.15	0	Male	68	G2	Stage IIIA	M0	N0
TCGA-DD-A113-01	79.66	0	Female	55	G3	Stage II	M0	N0
TCGA-DD-A114-01	37.75	1	Male	42	G3	Stage II	M0	NA
TCGA-DD-A115-01	83.51	1	Male	53	G2	Stage IIIA	M0	N0
TCGA-DD-A116-01	53.29	1	Male	68	G3	Stage IIIA	M0	N0
TCGA-DD-A118-01	112.91	0	Female	77	G2	Stage II	M0	N0
TCGA-DD-A119-01	7.33	1	Male	40	G3	Stage IV	M1	N0
TCGA-DD-A11A-01	2.6	0	Male	67	G3	Stage I	M0	N0
TCGA-DD-A11B-01	0.46	1	Male	73	G2	Stage I	M0	N0
TCGA-DD-A11C-01	21.75	0	Male	69	G3	Stage I	M0	N0
TCGA-DD-A11D-01	51.25	1	Female	57	G2	Stage I	M0	N0
TCGA-DD-A1E9-01	90.64	1	Male	70	G2	Stage I	M0	N0
TCGA-DD-A1EA-01	79.34	0	Male	68	G2	Stage II	M0	N0
TCGA-DD-A1EB-01	66.26	0	Female	72	G2	Stage I	M0	N0
TCGA-DD-A1EC-01	19.78	0	Female	20	G3	Stage I	M0	N0
TCGA-DD-A1ED-01	75.59	0	Male	68	G1	Stage I	M0	N0
TCGA-DD-A1EE-01	11.47	1	Male	73	G3	Stage IIIA	M0	N0
TCGA-DD-A1EF-01	12.94	1	Female	57	G3	Stage I	M0	N0
TCGA-DD-A1EG-01	45.07	1	Male	76	G3	Stage I	M0	N0
TCGA-DD-A1EH-01	49.11	0	Male	23	G3	Stage III	M0	N0
TCGA-DD-A1EI-01	6.01	0	Male	46	G2	Stage I	M0	N0

TCGA-DD-A1EJ-01	33.02	1	Female	71	G2	Stage IIIC	M0	N1
TCGA-DD-A1EK-01	18.33	1	Female	64	G2	Stage IVB	M1	N0
TCGA-DD-A1EL-01	13.63	1	Male	23	G3	Stage II	M0	N0
TCGA-DD-A39V-01	21.12	1	Male	77	G3	Stage II	M0	NX
TCGA-DD-A39W-01	27.17	1	Female	29	G2	Stage III	M0	N0
TCGA-DD-A39X-01	55.65	1	Female	78	G2	Stage I	M0	NX
TCGA-DD-A39Y-01	5.62	1	Male	67	G3	Stage I	M0	NX
TCGA-DD-A39Z-01	19.74	1	Female	43	G2	Stage II	M0	NX
TCGA-DD-A3A0-01	25.79	1	Male	70	G2	Stage I	M0	NX
TCGA-DD-A3A1-01	7.65	1	Male	65	G2	Stage IIIA	M0	N0
TCGA-DD-A3A2-01	70.01	1	Female	76	G1	Stage I	M0	N0
TCGA-DD-A3A3-01	17.58	1	Male	45	G2	Stage I	M0	N0
TCGA-DD-A3A4-01	20.11	1	Male	37	G3	Stage IIIA	M0	N0
TCGA-DD-A3A5-01	102.66	1	Female	66	G2	Stage III	M0	N0
TCGA-DD-A3A6-01	107.03	1	Female	72	G2	Stage II	M0	N0
TCGA-DD-A3A7-01	13.76	1	Male	67	G3	Stage IIIB	M0	N0
TCGA-DD-A3A8-01	0.36	1	Male	75	G2	Stage II	M0	N0
TCGA-DD-A3A9-01	30.58	1	Female	64	G2	Stage IVB	M1	N0
TCGA-DD-A4NA-01	33.11	0	Female	67	G3	Stage IIIC	M0	N1
TCGA-DD-A4NB-01	32.49	0	Male	25	G2	Stage I	M0	N0
TCGA-DD-A4ND-01	90.21	0	Female	56	G3	Stage I	M0	N0
TCGA-DD-A4NE-01	21.68	1	Female	75	G3	Stage IIIA	M0	N0
TCGA-DD-A4NF-01	30.95	0	Male	72	G2	Stage I	M0	N0
TCGA-DD-A4NG-01	26.35	1	Male	77	G2	Stage IIIA	M0	NX
TCGA-DD-A4NH-01	30.12	0	Female	65	G3	Stage IIIB	M0	N0
TCGA-DD-A4NI-01	26.81	0	Male	67	G2	Stage II	M0	NX
TCGA-DD-A4NJ-01	30.49	0	Female	54	G2	Stage II	M0	N0
TCGA-DD-A4NK-01	39.75	1	Female	80	G2	Stage IIIA	M0	N0
TCGA-DD-A4NL-01	56.21	0	Male	46	G1	Stage I	M0	N0
TCGA-DD-A4NN-01	29.53	1	Female	56	G3	Stage I	M0	N0
TCGA-DD-A4NO-01	73.75	0	Male	65	G1	Stage I	M0	N0
TCGA-DD-A4NP-01	108.67	0	Male	32	G3	Stage I	M0	N0
TCGA-DD-A4NQ-01	12.25	1	Male	60	G3	Stage II	M0	N0
TCGA-DD-A4NR-01	0.3	1	Female	85	G3	Stage I	M0	N0
TCGA-DD-A4NS-01	80.68	1	Female	61	G2	Stage I	M0	N0
TCGA-DD-A4NV-01	78.78	0	Male	61	G1	Stage IIIA	M0	N0
TCGA-DD-A73A-01	23.92	0	Male	71	G2	Stage I	M0	N0
TCGA-DD-A73B-01	9.3	1	Female	72	G2	Stage I	M0	N0
TCGA-DD-A73C-01	23.03	0	Female	65	G1	Stage IIIA	M0	N0
TCGA-DD-A73D-01	22.77	0	Female	68	G1	Stage II	MX	NX
TCGA-DD-A73E-01	1.45	0	Male	66	G1	Stage I	M0	N0
TCGA-DD-A73F-01	35.64	0	Female	77	G1	Stage I	M0	N0
TCGA-DD-A73G-01	114.26	0	Female	73	G3	Stage I	M0	N0
TCGA-DD-AA3A-01	13.47	1	Female	81	G4	Stage I	MX	N0
TCGA-DD-AAC8-01	0.53	1	Male	72	G3	Stage I	M0	N0
TCGA-DD-AAC9-01	11.4	0	Male	51	G2	Stage I	M0	N0
TCGA-DD-AACA-01	75.59	0	Male	65	G3	Stage I	M0	N0
TCGA-DD-AACA-02	75.59	0	Male	65	G3	Stage I	M0	N0
TCGA-DD-AACB-01	76.35	0	Female	74	G3	Stage I	M0	N0
TCGA-DD-AACC-01	55.35	1	Male	61	G2	Stage I	M0	N0
TCGA-DD-AACD-01	12.52	1	Male	48	G4	Stage I	M0	N0
TCGA-DD-AACE-01	71.75	0	Male	62	G3	Stage I	M0	N0
TCGA-DD-AACF-01	11.99	1	Male	68	G3	Stage I	M0	N0

TCGA-DD-AACG-01	15.41	1	Male	52	G4	Stage II	M0	N0
TCGA-DD-AACH-01	6.41	1	Male	69	G3	Stage II	M0	N0
TCGA-DD-AACI-01	53.15	0	Male	69	G3	Stage II	M0	N0
TCGA-DD-AACJ-01	69.05	0	Male	75	G2	Stage II	M0	N0
TCGA-DD-AACK-01	0.3	0	Male	70	G2	Stage I	M0	N0
TCGA-DD-AACL-01	3.52	1	Female	66	G3	Stage I	M0	N0
TCGA-DD-AACM-01	58.11	0	Male	48	G3	Stage II	M0	N0
TCGA-DD-AACN-01	42.77	0	Male	32	G3	Stage I	M0	N0
TCGA-DD-AACO-01	61.63	0	Male	40	G3	Stage I	M0	N0
TCGA-DD-AACP-01	13.63	0	Male	64	G3	Stage I	M0	N0
TCGA-DD-AACQ-01	14.19	1	Male	50	G3	Stage II	M0	N0
TCGA-DD-AACS-01	59.26	0	Male	39	G3	Stage I	M0	N0
TCGA-DD-AACT-01	51.31	0	Female	69	G2	Stage I	M0	N0
TCGA-DD-AACU-01	51.48	0	Male	59	G3	Stage I	M0	N0
TCGA-DD-AACV-01	50.3	0	Male	53	G3	Stage I	M0	N0
TCGA-DD-AACW-01	46.78	0	Male	43	G3	Stage I	M0	N0
TCGA-DD-AACX-01	5.58	0	Male	66	G3	Stage II	M0	N0
TCGA-DD-AACY-01	47.63	0	Male	61	G3	Stage I	M0	N0
TCGA-DD-AACZ-01	5.62	1	Female	63	G4	Stage I	M0	N0
TCGA-DD-AAD0-01	4.5	0	Female	73	G2	Stage I	M0	N0
TCGA-DD-AAD1-01	18.53	0	Female	51	G4	Stage I	M0	N0
TCGA-DD-AAD2-01	21.62	0	Male	66	G2	Stage I	M0	N0
TCGA-DD-AAD3-01	42.54	0	Male	43	G2	Stage I	M0	N0
TCGA-DD-AAD5-01	44.19	0	Male	54	G3	Stage I	M0	N0
TCGA-DD-AAD6-01	22.08	0	Male	66	G3	Stage IIIA	M0	N0
TCGA-DD-AAD8-01	40.05	0	Female	73	G2	Stage I	M0	N0
TCGA-DD-AADA-01	40.51	0	Female	66	G3	Stage I	M0	N0
TCGA-DD-AADB-01	40.8	0	Male	51	G4	Stage I	M0	N0
TCGA-DD-AADC-01	13.96	1	Male	53	G3	Stage I	M0	N0
TCGA-DD-AADD-01	40.44	0	Male	51	G4	Stage I	M0	N0
TCGA-DD-AADE-01	39.49	0	Male	50	G4	Stage I	M0	N0
TCGA-DD-AADF-01	3.78	1	Female	64	G4	Stage I	M0	N0
TCGA-DD-AADG-01	37.61	0	Male	70	G3	Stage IIIA	M0	N0
TCGA-DD-AADI-01	35.64	0	Female	43	G3	Stage I	M0	N0
TCGA-DD-AADJ-01	35.02	0	Female	70	G3	Stage I	M0	N0
TCGA-DD-AADK-01	34.46	0	Female	68	G3	Stage II	M0	N0
TCGA-DD-AADL-01	20.89	0	Male	58	G4	Stage I	M0	N0
TCGA-DD-AADM-01	0.39	1	Male	58	G3	Stage II	M0	N0
TCGA-DD-AADN-01	29.5	0	Male	59	G4	Stage I	MX	NX
TCGA-DD-AADO-01	14.88	0	Male	55	G3	Stage I	M0	N0
TCGA-DD-AADP-01	15.05	0	Male	45	G3	Stage I	M0	N0
TCGA-DD-AADQ-01	14.32	0	Male	59	G3	Stage II	M0	N0
TCGA-DD-AADR-01	66.62	0	Male	58	G3	Stage I	M0	N0
TCGA-DD-AADS-01	15.57	0	Male	63	G2	Stage I	M0	N0
TCGA-DD-AADU-01	18.2	0	Male	60	G3	Stage II	M0	N0
TCGA-DD-AADV-01	18.86	0	Male	50	G3	Stage I	M0	N0
TCGA-DD-AADW-01	19.28	0	Male	48	G3	Stage I	M0	N0
TCGA-DD-AADY-01	18.23	0	Female	55	G2	Stage I	M0	N0
TCGA-DD-AAE0-01	18.23	0	Female	45	G4	Stage IIIA	M0	N0
TCGA-DD-AAE1-01	18.13	0	Male	52	G3	Stage I	M0	N0
TCGA-DD-AAE2-01	20.96	0	Male	51	G3	Stage I	M0	N0
TCGA-DD-AAE3-01	18.59	0	Male	50	G2	Stage I	M0	N0
TCGA-DD-AAE4-01	19.97	0	Female	49	G1	Stage I	M0	N0

TCGA-DD-AAE6-01	4.63	0	Female	59	G2	Stage I	M0	N0
TCGA-DD-AAE7-01	21.16	0	Male	72	G2	Stage I	M0	N0
TCGA-DD-AAE8-01	21.81	0	Male	45	G3	Stage I	M0	N0
TCGA-DD-AAE9-01	23.72	0	Male	69	G3	Stage I	M0	N0
TCGA-DD-AAEA-01	18.89	0	Male	65	G3	Stage I	M0	N0
TCGA-DD-AAEB-01	15.7	0	Male	60	G2	Stage I	M0	N0
TCGA-DD-AAED-01	25.07	0	Male	51	G3	Stage I	M0	N0
TCGA-DD-AAEE-01	26.61	0	Male	55	G4	Stage I	M0	N0
TCGA-DD-AAEG-01	23.62	0	Female	59	G3	Stage I	M0	N0
TCGA-DD-AAEH-01	25.76	0	Male	73	G2	Stage I	M0	N0
TCGA-DD-AAEI-01	50.3	0	Male	72	G2	Stage I	M0	N0
TCGA-DD-AAEK-01	35.05	0	Male	51	G3	Stage II	M0	N0
TCGA-DD-AAVP-01	90.41	0	Male	48	G1	Stage I	M0	N0
TCGA-DD-AAVQ-01	89.62	0	Male	38	G2	Stage I	M0	N0
TCGA-DD-AAVR-01	82.56	0	Male	44	G2	Stage I	M0	N0
TCGA-DD-AAVS-01	59.89	0	Male	56	G2	Stage I	M0	N0
TCGA-DD-AAVU-01	72.34	0	Male	46	G2	Stage II	M0	N0
TCGA-DD-AAVV-01	80.65	0	Male	56	G3	Stage II	M0	N0
TCGA-DD-AAVW-01	76.12	0	Male	35	G2	Stage I	M0	N0
TCGA-DD-AAVX-01	56.44	0	Male	38	G2	Stage II	M0	N0
TCGA-DD-AAVY-01	64.72	0	Male	56	G2	Stage IIIA	M0	N0
TCGA-DD-AAVZ-01	62.42	0	Male	38	G2	Stage I	M0	N0
TCGA-DD-AAW0-01	66.2	0	Male	54	G2	Stage I	M0	N0
TCGA-DD-AAW1-01	65.34	0	Male	55	G2	Stage IIIA	M0	N0
TCGA-DD-AAW2-01	60.94	0	Male	69	G2	Stage I	M0	N0
TCGA-DD-AAW3-01	53.65	0	Male	69	G2	Stage I	M0	N0
TCGA-ED-A459-01	29.89	0	Male	47	G2	Stage II	M0	N0
TCGA-ED-A4XI-01	26.91	0	Male	58	G3	Stage II	M0	N0
TCGA-ED-A5KG-01	28.06	0	Female	60	G2	Stage II	M0	N0
TCGA-ED-A627-01	13.9	0	Male	74	G2	Stage I	M0	NX
TCGA-ED-A66X-01	13.34	0	Male	35	G3	Stage IIIA	M0	N0
TCGA-ED-A66Y-01	9.72	1	Female	51	G3	Stage IIIA	M0	N0
TCGA-ED-A7PX-01	0.2	0	Female	48	G3	Stage II	M0	NX
TCGA-ED-A7PY-01	12.81	0	Female	20	G3	Stage II	M0	NX
TCGA-ED-A7PZ-01	0.2	0	Male	61	G2	Stage II	M0	NX
TCGA-ED-A7XO-01	14.03	0	Male	29	G2	Stage IIIA	M0	N0
TCGA-ED-A7XP-01	13.14	0	Female	53	G3	Stage II	M0	N0
TCGA-ED-A82E-01	13.4	0	Female	60	G2	Stage IIIA	M0	N0
TCGA-ED-A8O5-01	13.34	0	Female	59	G3	Stage IIIA	M0	N0
TCGA-ED-A8O6-01	1.84	1	Female	50	G3	Stage IIIA	M0	N0
TCGA-ED-A97K-01	0.2	0	Male	54	G2	Stage IIIA	M0	N0
TCGA-EP-A12J-01	18.73	0	Male	62	G1	Stage I	MX	NX
TCGA-EP-A26S-01	19.97	0	Male	70	G2	Stage I	MX	N0
TCGA-EP-A2KA-01	20.6	1	Female	52	G3	Stage IIIA	MX	NX
TCGA-EP-A2KB-01	19.58	1	Female	46	G2	Stage I	MX	NX
TCGA-EP-A2KC-01	0.62	1	Male	62	G3	Stage I	MX	NX
TCGA-EP-A3JL-01	9.95	0	Male	76	G2	Stage I	MX	NX
TCGA-EP-A3RK-01	11.93	0	Male	73	G2	Stage IIIA	MX	NX
TCGA-ES-A2HS-01	22.6	1	Male	80	G2	Stage I	MX	NX
TCGA-ES-A2HT-01	14.39	1	Male	54	G2	Stage I	MX	NX
TCGA-FV-A23B-01	60.84	1	Female	70	NA	Stage II	M0	N0
TCGA-FV-A2QQ-01	23.95	0	Male	80	G2	Stage I	MX	N0
TCGA-FV-A2QR-01	19.09	1	Male	75	G1	Stage I	M0	N0

TCGA-FV-A3I0-01	27.86	0	Female	76	G2	Stage II	M0	NX
TCGA-FV-A3I1-01	8.11	1	Female	81	G2	Stage II	MX	N0
TCGA-FV-A3R2-01	6.37	1	Male	75	NA	Stage I	MX	NX
TCGA-FV-A3R3-01	12.02	1	Female	38	G2	Stage I	MX	NX
TCGA-FV-A495-01	0.03	0	Female	51	G2	Stage II	M0	NX
TCGA-FV-A496-01	0.33	0	Female	84	G2	Stage I	M0	NX
TCGA-FV-A4ZP-01	81.67	1	Male	78	G2	Stage IIIA	M0	NX
TCGA-FV-A4ZQ-01	0.39	0	Male	52	G2	Stage I	M0	NX
TCGA-G3-A25S-01	13.67	1	Male	64	G2	Stage I	M0	N0
TCGA-G3-A25T-01	51.02	0	Female	45	G2	Stage IIIA	M0	N0
TCGA-G3-A25U-01	53.75	0	Female	63	G3	Stage I	M0	N0
TCGA-G3-A25V-01	28.25	0	Male	68	G2	Stage I	M0	N0
TCGA-G3-A25W-01	30.72	0	Female	79	G2	Stage IIIB	M0	N0
TCGA-G3-A25X-01	58.44	0	Male	73	G3	Stage II	M0	N0
TCGA-G3-A25Y-01	14.85	1	Female	52	G3	Stage I	M0	N0
TCGA-G3-A25Z-01	21.52	0	Male	58	G2	Stage I	M0	N0
TCGA-G3-A3CG-01	22.11	0	Male	80	G2	Stage I	M0	N0
TCGA-G3-A3CH-01	25.62	0	Male	53	G2	Stage IIIA	M0	N0
TCGA-G3-A3CI-01	5.91	0	Male	71	G2	Stage I	M0	N0
TCGA-G3-A3CJ-01	19.51	0	Male	52	G2	Stage II	M0	N0
TCGA-G3-A3CK-01	19.22	0	Male	61	G2	Stage I	M0	N0
TCGA-G3-A5SI-01	25.23	1	Male	44	G2	Stage II	M0	N0
TCGA-G3-A5SJ-01	22.93	0	Male	59	G2	Stage I	M0	NX
TCGA-G3-A5SK-01	24.44	0	Male	58	G1	Stage I	M0	NX
TCGA-G3-A5SL-01	20.4	0	Male	70	G2	Stage II	M0	NX
TCGA-G3-A5SM-01	17.08	0	Male	58	G3	Stage II	M0	NX
TCGA-G3-A6UC-01	22.04	0	Male	65	G2	Stage IIIB	M0	N0
TCGA-G3-A7M5-01	14.68	0	Male	76	G2	Stage I	MX	NX
TCGA-G3-A7M6-01	20.76	0	Female	60	G3	Stage I	MX	NX
TCGA-G3-A7M7-01	11.86	0	Male	65	G1	Stage I	MX	NX
TCGA-G3-A7M8-01	14.13	0	Male	31	G1	Stage I	MX	NX
TCGA-G3-A7M9-01	1.84	1	Male	70	G2	Stage IIIB	MX	NX
TCGA-G3-AAUZ-01	15.77	0	Male	48	G2	Stage I	M0	N0
TCGA-G3-AAV0-01	15.64	0	Male	58	G2	Stage I	M0	N0
TCGA-G3-AAV1-01	11.79	1	Male	51	G3	Stage IIIC	M0	N0
TCGA-G3-AAV2-01	12.22	0	Male	50	G1	Stage I	M0	N0
TCGA-G3-AAV3-01	13.53	0	Female	58	G2	Stage II	M0	N0
TCGA-G3-AAV4-01	0.89	1	Female	83	G1	Stage I	M0	N0
TCGA-G3-AAV5-01	11.63	0	Male	67	G2	Stage II	M0	N0
TCGA-G3-AAV6-01	2.14	1	Female	53	G3	Stage IIIA	M0	N0
TCGA-G3-AAV7-01	11.86	0	Male	38	G2	Stage II	M0	N0
TCGA-GJ-A3OU-01	28.88	0	Male	59	G2	Stage I	MX	NX
TCGA-GJ-A6C0-01	1.02	1	Female	75	G2	Stage II	MX	NX
TCGA-GJ-A9DB-01	2.2	1	Male	68	G2	Stage I	MX	N0
TCGA-HP-A5MZ-01	2.99	1	Male	78	G2	Stage I	M0	NX
TCGA-HP-A5N0-01	37.68	1	Female	90	NA	NA	M0	NX
TCGA-K7-A5RF-01	20.73	0	Male	64	G1	Stage I	MX	NX
TCGA-K7-A5RG-01	17.05	0	Male	66	G1	Stage I	MX	NX
TCGA-K7-A6G5-01	16.82	0	Male	66	G2	Stage I	MX	N0
TCGA-K7-AAU7-01	11.79	0	Male	61	G2	Stage II	MX	NX
TCGA-KR-A7K0-01	2.14	1	Male	65	G1	Stage I	M0	N0
TCGA-KR-A7K2-01	27.23	0	Male	64	G1	Stage I	M0	N0
TCGA-KR-A7K7-01	31.24	0	Female	61	G1	Stage II	M0	N0

TCGA-KR-A7K8-01	29.76	0	Male	57	G1	Stage I	M0	N0
TCGA-LG-A6GG-01	12.71	0	Female	79	G2	Stage II	M0	NX
TCGA-LG-A9QC-01	13.96	0	Male	48	G2	Stage I	M0	NX
TCGA-LG-A9QD-01	12.02	0	Male	68	G2	Stage IIIA	M0	N0
TCGA-MI-A75C-01	9.56	0	Male	64	G3	Stage I	M0	N0
TCGA-MI-A75E-01	16.66	0	Male	61	G2	Stage IIIC	M0	N0
TCGA-MI-A75G-01	22.93	0	Male	63	G2	Stage II	M0	N0
TCGA-MI-A75H-01	24.54	0	Male	77	NA	NA	MX	NX
TCGA-MI-A75I-01	20.7	0	Male	61	G1	NA	MX	NX
TCGA-MR-A520-01	7.52	0	Male	58	G1	Stage I	MX	NX
TCGA-MR-A8JO-01	10.84	0	Male	34	G3	Stage I	MX	N0
TCGA-NI-A4U2-01	58.84	1	Male	71	G1	Stage IIIA	MX	NX
TCGA-NI-A8LF-01	26.25	0	Male	74	G3	Stage I	MX	NX
TCGA-O8-A75V-01	17.67	0	Male	54	G2	Stage I	MX	NX
TCGA-PD-A5DF-01	20.99	1	Female	58	G2	Stage IIIB	M0	N0
TCGA-QA-A7B7-01	3.09	0	Male	48	G2	Stage II	MX	NX
TCGA-RC-A6M4-01	0.72	0	Female	74	G2	Stage IIIA	MX	NX
TCGA-RC-A6M5-01	0.49	0	Female	20	G2	Stage IVA	M0	N1
TCGA-RC-A6M6-01	0.3	0	Male	75	G3	Stage II	M0	NX
TCGA-RC-A7S9-01	21.02	0	Female	47	G3	Stage I	M0	N0
TCGA-RC-A7SB-01	19.32	0	Male	53	G2	Stage II	M0	N0
TCGA-RC-A7SF-01	19.02	0	Male	66	G2	Stage I	M0	N0
TCGA-RC-A7SH-01	15.37	0	Male	42	G3	Stage II	M0	N0
TCGA-RC-A7SK-01	15.51	0	Male	59	G3	Stage I	M0	N0
TCGA-RG-A7D4-01	36.07	0	Male	69	G2	Stage II	M0	N0
TCGA-T1-A6J8-01	0.76	0	Male	68	G2	NA	M0	NX
TCGA-UB-A7MA-01	27.86	0	Female	62	G2	Stage II	M0	N0
TCGA-UB-A7MB-01	19.74	0	Male	24	G3	Stage II	MX	NX
TCGA-UB-A7MC-01	16.43	0	Male	59	G3	Stage IIIA	MX	N0
TCGA-UB-A7MD-01	1.71	1	Male	67	G3	Stage I	MX	N0
TCGA-UB-A7ME-01	15.97	0	Male	51	G2	Stage I	MX	NX
TCGA-UB-A7MF-01	7.03	1	Male	56	G2	Stage IIIA	MX	NX
TCGA-UB-AA0U-01	10.74	0	Male	60	G2	Stage II	MX	NX
TCGA-UB-AA0V-01	10.32	0	Female	69	G1	Stage I	MX	NX
TCGA-WJ-A86L-01	11.33	0	Female	68	G2	Stage I	MX	NX
TCGA-WQ-A9G7-01	0.99	0	Female	71	G3	NA	M0	NX
TCGA-WQ-AB4B-01	12.98	0	Male	62	G2	Stage II	M0	NX
TCGA-WX-AA44-01	20.2	0	Female	64	G3	Stage I	MX	NX
TCGA-WX-AA46-01	24.84	0	Male	61	G1	Stage II	MX	NX
TCGA-WX-AA47-01	18.27	1	Female	33	G2	Stage IIIA	MX	NX
TCGA-XR-A8TC-01	43.99	0	Female	43	G2	Stage I	MX	NX
TCGA-XR-A8TD-01	33.84	0	Female	49	G3	Stage IIIB	M0	N0
TCGA-XR-A8TE-01	30.39	0	Male	16	G1	Stage IIIA	MX	N0
TCGA-XR-A8TF-01	22.77	1	Male	74	G1	Stage I	MX	NX
TCGA-XR-A8TG-01	29.5	0	Male	58	G2	Stage I	M0	NX
TCGA-YA-A8S7-01	13.53	1	Male	68	G3	Stage IIIA	MX	N0
TCGA-ZP-A9CV-01	35.74	1	Male	59	G1	NA	MX	NX
TCGA-ZP-A9CY-01	25.69	0	Female	66	G1	NA	MX	NX
TCGA-ZP-A9CZ-01	23.19	0	Male	72	G1	NA	MX	NX
TCGA-ZP-A9D0-01	35.84	0	Female	67	G1	NA	MX	NX
TCGA-ZP-A9D1-01	0.69	0	Female	56	G2	NA	MX	NX
TCGA-ZP-A9D2-01	25.13	1	Male	51	G2	NA	MX	NX
TCGA-ZP-A9D4-01	12.98	0	Female	64	G1	NA	MX	NX

TCGA-ZS-A9CD-01	45.53	1	Male	73	G2	Stage II	MX	NX
TCGA-ZS-A9CE-01	40.77	0	Female	79	G1	Stage II	MX	NX
TCGA-ZS-A9CF-01	79.24	0	Male	64	G2	Stage II	MX	NX
TCGA-ZS-A9CF-02	79.24	0	Male	64	G2	Stage II	MX	NX
TCGA-ZS-A9CG-01	11.2	0	Male	55	G2	Stage II	MX	NX

T
T2
T1
T1
T1
T1
T1
T2
T2
T3
T1
T1
T2
T1
T1
T1
T1
T1
T1
T2
T1
T3b
T2
T2
T1
T2
T2
T3a
T2
T3
T3
T2
T3
T3
T4
T2
T4
T3a
T4
T1
T1
T3
T3
T2
T1
T2
T3a
T2
T1
T4
T2

T1
T2
T3a
T2
T4
T4
T2
T4
T3
T3
T3
T3
T3
T3
T3
T3
T3
T3
T4
T3
T3
T2
T3
T3
T2
T3
T3
T3
T3
T3
T2
T2
T3
T2
T2
T3
T3
T2
T3a
T1
T1
T1
T1
T1
T2
T1
T1
T1
T3
T1
T1
T3
T1

T1
T4
T2
T2
T3
T1
T1
T2
T1
T3b
T1
T1
T3
T3
T2
T3b
T2
T4
T2
T1
T1
T3a
T1
T3a
T3b
T2
T2
T3
T1
T1
T1
T1
T2
T1
T1
T3
T1
T1
T3a
T2
T1
T1
T1
T1
T1
T1
T1
T1
T1
T1
T1
T1
T1

T2
T2
T2
T2
T1
T1
T2
T1
T1
T1
T2
T1
T1
T1
T1
T1
T2
T1
T1
T1
T1
T1
T1
T1
T3a
T1
T1
T1
T1
T1
T1
T1
T3a
T1
T1
T2
T1
T2
T1
T1
T1
T2
T1
T1
T2
T1
T1
T1
T3a
T1
T1
T1
T1

T1
T1
T1
T1
T1
T1
T1
T1
T1
T1
T1
T2
T1
T1
T1
T1
T2
T2
T1
T2
T3
T1
T1
T3
T1
T1
T2
T2
T2
T1
T3a
T3a
T2
T2
T2
T3a
T2
T3a
T3a
T3a
T3a
T1
T1
T3a
T1
T1
T1
T3a
T1
T1
T2
T1
T1

T2
T2
T1
T1
T2
T1
T3
T1
T1
T3
T1
T1
T3b
T2
T1
T1
T1
T3a
T1
T2
T1
T2
T1
T1
T2
T2
T3b
T1
T1
T1
T1
T3b
T1
T1
T4
T1
T2
T1
T2
T3a
T2
T1
T2
T1
T1
TX
T1
T1
T1
T2a
T1
T1
T2

T1
T2
T1
T3a
T1
T4
T2
NA
T2
T1
T1
T3
T1
T1
T4
T2
T3
T1
T2
T1
T2
T1
T2
T1
T2
T1
T2b
T2
T3a
T1
T1
T3a
T2
NA
T1
T3a
T2
T1
T2
T3a
T1
T3
T3
T1
T1
T3a
T1
T1
T1
T1
T1
T2
T1

T2
T2
T2
T2
T2
