# Supplementary material for: M6A Modifier-Mediated Methylation Characterized by Diverse Prognosis, Tumor Microenvironment, and Immunotherapy Response in Hepatocellular Carcinoma
Source: J Oncol. 2022 Aug 16;2022:2513813. doi: 10.1155/2022/2513813 (PMC9398803; doi:10.1155/2022/2513813)
Supplement: Supplementary Materials — Supplementary Figure 1. Consensus clustering analyses of stratifying HCC cases in TCGA cohort into three m6A methylation patterns according to 23 m6A regulators. (A) Heatmap for the consensus matrix k = 3. (B) Cumulative distribution function (CDF) under diverse k values. (C) Delta area diagram for relative alterations in area under CDF curves. (D) The tracking plot for HCC samples under different k values. Supplementary Figure 2. Consensus clustering analyses for clustering three m6A genomic phenotypes in the light of the expression profiling of m6A-associated genes in TCGA cohort. (A) Heatmap for the consensus matrix k = 3. (B) CDF under diverse k values. (C) Delta area diagram for relative alterations in area under CDF curves. (D) The tracking plot for HCC samples under different k values. Supplementary Figure 3. Subgroup analysis of the prognosis value of m6A score among HCC patients in TCGA data set. Kaplan-Meir curves of cases with high or low m6A score in each subgroup: (A) age ≥ 65; (B) age < 65; (C) female; (D) male; (E) G1-2; (F) G3-4; (G) stage I-II; (H) stage III-IV. P values were determined through log-rank tests. Supplementary Table 1. The clinical information of HCC samples in the TCGA data set. Supplementary Table 2. The clinical information of HCC samples in the GSE14520 data set. Supplementary Table 3. The list of 331 m6A phenotype-associated DEGs. [file 2513813.f1.zip › 2513813.f1/Supplementary table 2.pdf]

Supplementary table 2. The clinical information of HCC samples in the GSE14520 dataset.

LCS ID	ID	Affy_GSM	Tissue Type	Predicted risk Metastasis Signature
LCS_193A	03-195	GSM363205	Tumor	low
LCS_094A	02-304	GSM363115	Tumor	low
LCS_085A	02-286	GSM362970	Tumor	low
LCS_207A	03-228	GSM363354	Tumor	high
LCS_272A	03-434	GSM363039	Tumor	low
LCS_021A	03-077	GSM363209	Tumor	low
LCS_137A	02-448	GSM363344	Tumor	low
LCS_268A	03-419	GSM363271	Tumor	high
LCS_046A	03-311	GSM363404	Tumor	low
LCS_250A	03-342	GSM363217	Tumor	high
LCS_211A	03-234	GSM363053	Tumor	low
LCS_048A	03-321	GSM363295	Tumor	low
LCS_222A	03-262	GSM363272	Tumor	low
LCS_047A	03-309	GSM363164	Tumor	low
LCS_095A	02-307	GSM362965	Tumor	high
LCS_179A	03-138	GSM363126	Tumor	high
LCS_210A	03-233	GSM363082	Tumor	low
LCS_143A	02-458	GSM363176	Tumor	low
LCS_033A	03-150	GSM363099	Tumor	high
LCS_056A	03-333	GSM363316	Tumor	low
LCS_199A	03-218	GSM363178	Tumor	high
LCS_116A	02-359	GSM363343	Tumor	low
LCS_150A	03-017	GSM363033	Tumor	high
LCS_230A	03-274	GSM363055	Tumor	high
LCS_164A	03-061	GSM363100	Tumor	high
LCS_182A	03-151	GSM363249	Tumor	high
LCS_333A	LCS-333	GSM363420	Tumor	high
LCS_254A	03-356	GSM362952	Tumor	low
LCS_078A	02-269	GSM363130	Tumor	low
LCS_156A	03-042	GSM363149	Tumor	low
LCS_341A	LCS-341	GSM363424	Tumor	low
LCS_106A	02-339	GSM363337	Tumor	high
LCS_274A	03-438	GSM363031	Tumor	low
LCS_075A	03-381	GSM363166	Tumor	low
LCS_152A	03-024	GSM363049	Tumor	high
LCS_159A	03-049	GSM363032	Tumor	high
LCS_183A	03-162	GSM363072	Tumor	low
LCS_415A	LCS-415	GSM363444	Tumor	high
LCS_066A	03-354	GSM363289	Tumor	low
LCS_424A	LCS-424	GSM363446	Tumor	low
LCS_012A	03-196	GSM363296	Tumor	high
LCS_206A	03-227	GSM363235	Tumor	high
LCS_079A	02-271	GSM362958	Tumor	low
LCS_289A	03-457	GSM363366	Tumor	low
LCS_051A	03-292	GSM363311	Tumor	low
LCS_180A	03-139	GSM363071	Tumor	low
LCS_266A	03-416	GSM363329	Tumor	low
LCS_044A	03-241	GSM363200	Tumor	low
LCS_130A	02-429	GSM362984	Tumor	low
LCS_339A	LCS-339	GSM363422	Tumor	high

LCS_162A	03-057	GSM363037	Tumor	high
LCS_269A	03-424	GSM363362	Tumor	low
LCS_251A	03-343	GSM363357	Tumor	high
LCS_069A	03-364	GSM363080	Tumor	high
LCS_160A	03-054	GSM363125	Tumor	high
LCS_171A	03-102	GSM363106	Tumor	high
LCS_135A	02-445	GSM362988	Tumor	low
LCS_249A	03-336	GSM363107	Tumor	low
LCS_231A	03-277	GSM363355	Tumor	high
LCS_256A	03-374	GSM363087	Tumor	low
LCS_228A	03-272	GSM363268	Tumor	high
LCS_177A	03-131	GSM363015	Tumor	high
LCS_023A	03-082	GSM363014	Tumor	low
LCS_273A	03-435	GSM363364	Tumor	low
LCS_014A	03-062	GSM363034	Tumor	low
LCS_344A	LCS-344	GSM363428	Tumor	high
LCS_015A	03-071	GSM363078	Tumor	high
LCS_174A	03-112	GSM363202	Tumor	low
LCS_032A	03-148	GSM363332	Tumor	high
LCS_165A	03-073	GSM363313	Tumor	low
LCS_035A	03-186	GSM363017	Tumor	high
LCS_148A	03-012	GSM363294	Tumor	high
LCS_167A	03-087	GSM363330	Tumor	high
LCS_343A	LCS-343	GSM363426	Tumor	high
LCS_263A	03-404	GSM363360	Tumor	low
LCS_188A	03-180	GSM363008	Tumor	high
LCS_253A	03-350	GSM363143	Tumor	high
LCS_426A	LCS-426	GSM363448	Tumor	low
LCS_134A	02-437	GSM363127	Tumor	high
LCS_245A	03-314	GSM363102	Tumor	low
LCS_090A	02-295	GSM363151	Tumor	low
LCS_074A	03-398	GSM363124	Tumor	low
LCS_190A	03-187	GSM712542	Tumor	high
LCS_031A	03-141	GSM363335	Tumor	low
LCS_142A	02-457	GSM363350	Tumor	high
LCS_197A	03-210	GSM363386	Tumor	high
LCS_215A	03-243	GSM363207	Tumor	high
LCS_123A	02-384	GSM362986	Tumor	high
LCS_103A	02-325	GSM362972	Tumor	low
LCS_184A	03-164	GSM363186	Tumor	low
LCS_025A	03-101	GSM363288	Tumor	low
LCS_140A	02-455	GSM363016	Tumor	low
LCS_109A	02-346	GSM363341	Tumor	high
LCS_236A	03-282	GSM363243	Tumor	low
LCS_259A	03-383	GSM363168	Tumor	low
LCS_209A	03-231	GSM363051	Tumor	high
LCS_213A	03-237	GSM363098	Tumor	high
LCS_208A	03-230	GSM363314	Tumor	low
LCS_264A	03-405	GSM363170	Tumor	low
LCS_132A	02-431	GSM363029	Tumor	high
LCS_039A	03-212	GSM362950	Tumor	high
LCS_260A	03-387	GSM363298	Tumor	high
LCS_117A	02-361	GSM363265	Tumor	high

LCS_104A	02-335	GSM363038	Tumor	low
LCS_247A	03-316	GSM363309	Tumor	high
LCS_061A	03-361	GSM363104	Tumor	low
LCS_216A	03-244	GSM363393	Tumor	high
LCS_120A	02-370	GSM363011	Tumor	high
LCS_034A	03-149	GSM363180	Tumor	low
LCS_237A	03-284	GSM363048	Tumor	low
LCS_275A	03-439	GSM363077	Tumor	low
LCS_279A	03-450	GSM363144	Tumor	high
LCS_016A	03-064	GSM363336	Tumor	low
LCS_401A	LCS-401	GSM363438	Tumor	high
LCS_153A	03-025	GSM363073	Tumor	high
LCS_099A	02-315	GSM362977	Tumor	low
LCS_286A	03-422	GSM363012	Tumor	low
LCS_240A	03-291	GSM363328	Tumor	low
LCS_084A	02-285	GSM363215	Tumor	low
LCS_238A	03-285	GSM363400	Tumor	low
LCS_072A	03-375	GSM363358	Tumor	low
LCS_201A	03-220	GSM363391	Tumor	low
LCS_050A	03-319	GSM363333	Tumor	high
LCS_265A	03-407	GSM363083	Tumor	high
LCS_011A	02-461	GSM363218	Tumor	high
LCS_169A	03-093	GSM363086	Tumor	low
LCS_100A	02-318	GSM363267	Tumor	low
LCS_136A	02-447	GSM362994	Tumor	low
LCS_194A	03-199	GSM363224	Tumor	low
LCS_054A	03-322	GSM363269	Tumor	low
LCS_126A	02-415	GSM363297	Tumor	high
LCS_278A	03-444	GSM363182	Tumor	low
LCS_045A	03-306	GSM363109	Tumor	high
LCS_347A	LCS-347	GSM363432	Tumor	low
LCS_073A	03-384	GSM363054	Tumor	high
LCS_018A	03-051	GSM363075	Tumor	low
LCS_224A	03-265	GSM363129	Tumor	high
LCS_223A	03-263	GSM363101	Tumor	high
LCS_161A	03-055	GSM363378	Tumor	high
LCS_195A	03-200	GSM363232	Tumor	low
LCS_127A	02-423	GSM362993	Tumor	low
LCS_282A	03-456	GSM363150	Tumor	high
LCS_093A	02-302	GSM363142	Tumor	low
LCS_086A	02-287	GSM363013	Tumor	high
LCS_028A	03-121	GSM363331	Tumor	low
LCS_009A	02-409	GSM363128	Tumor	high
LCS_110A	02-347	GSM363315	Tumor	low
LCS_406A	LCS-406	GSM363442	Tumor	high
LCS_172A	03-105	GSM363384	Tumor	high
LCS_291A	AN	GSM363147	Tumor	high
LCS_290A	03-469	GSM363368	Tumor	low
LCS_005A	02-407	GSM362947	Tumor	low
LCS_234A	03-280	GSM363081	Tumor	high
LCS_065A	03-363	GSM363052	Tumor	high
LCS_102A	02-323	GSM362966	Tumor	low
LCS_010A	02-424	GSM363169	Tumor	high

LCS_040A	03-215	GSM363085	Tumor	high
LCS_200A	03-219	GSM363388	Tumor	high
LCS_043A	03-208	GSM363196	Tumor	low
LCS_019A	03-050	GSM362948	Tumor	low
LCS_267A	03-418	GSM363266	Tumor	low
LCS_147A	02-466	GSM363326	Tumor	high
LCS_261A	03-394	GSM363230	Tumor	low
LCS_036A	03-214	GSM363237	Tumor	high
LCS_166A	03-081	GSM363204	Tumor	high
LCS_125A	02-396	GSM362992	Tumor	low
LCS_277A	03-443	GSM363264	Tumor	low
LCS_243A	03-303	GSM363312	Tumor	low
LCS_057A	03-326	GSM363123	Tumor	low
LCS_038A	03-213	GSM363245	Tumor	high
LCS_284A	03-467	GSM363192	Tumor	low
LCS_121A	02-375	GSM363291	Tumor	low
LCS_192A	03-192	GSM363273	Tumor	low
LCS_281A	03-453	GSM363148	Tumor	high
LCS_189A	03-182	GSM363263	Tumor	high
LCS_105A	02-337	GSM362978	Tumor	low
LCS_262A	03-397	GSM363056	Tumor	high
LCS_088A	02-290	GSM363310	Tumor	high
LCS_175A	03-125	GSM363275	Tumor	low
LCS_196A	03-205	GSM363352	Tumor	high
LCS_157A	03-043	GSM363293	Tumor	low
LCS_027A	03-119	GSM363292	Tumor	high
LCS_129A	02-428	GSM363211	Tumor	high
LCS_068A	03-369	GSM362954	Tumor	high
LCS_212A	03-235	GSM363070	Tumor	high
LCS_107A	02-344	GSM363339	Tumor	high
LCS_063A	03-346	GSM363270	Tumor	low
LCS_097A	02-313	GSM363371	Tumor	low
LCS_185A	03-165	GSM363009	Tumor	high
LCS_122A	02-380	GSM362982	Tumor	high
LCS_203A	03-223	GSM363188	Tumor	low
LCS_002A	02-395	GSM363251	Tumor	low
LCS_144A	02-459	GSM363057	Tumor	high
LCS_205A	03-226	GSM363030	Tumor	high
LCS_022A	03-099	GSM363274	Tumor	high
LCS_049A	03-317	GSM363146	Tumor	high
LCS_004A	02-404	GSM362983	Tumor	low
LCS_219A	03-255	GSM363222	Tumor	low
LCS_042A	03-206	GSM363074	Tumor	high
LCS_400A	LCS-400	GSM363436	Tumor	high
LCS_248A	03-334	GSM363290	Tumor	high
LCS_007A	02-421	GSM362987	Tumor	low
LCS_227A	03-270	GSM363035	Tumor	low
LCS_283A	03-460	GSM363036	Tumor	low
LCS_118A	02-363	GSM363079	Tumor	high
LCS_146A	02-462	GSM363317	Tumor	high
LCS_163A	03-058	GSM363172	Tumor	high
LCS_149A	03-015	GSM363084	Tumor	low
LCS_145A	02-460	GSM363239	Tumor	high

LCS_131A	02-430	GSM363121	Tumor	high
LCS_020A	03-070	GSM363108	Tumor	low
LCS_151A	03-023	GSM363152	Tumor	low
LCS_191A	03-188	GSM363105	Tumor	high
LCS_173A	03-108	GSM363327	Tumor	low
LCS_108A	02-345	GSM363194	Tumor	high
LCS_138A	02-452	GSM363346	Tumor	low
LCS_403A	LCS-403	GSM363440	Tumor	high
LCS_089A	02-291	GSM363174	Tumor	high
LCS_091A	02-298	GSM362976	Tumor	low
LCS_178A	03-133	GSM363190	Tumor	high
LCS_024A	03-088	GSM363069	Tumor	high
LCS_270A	03-425	GSM363247	Tumor	low
LCS_062A	03-345	GSM363076	Tumor	high
LCS_158A	03-047	GSM363376	Tumor	low
LCS_092A	02-301	GSM362959	Tumor	high
LCS_170A	03-098	GSM363226	Tumor	low
LCS_154A	03-033	GSM363010	Tumor	high
LCS_071A	03-370	GSM363220	Tumor	high
LCS_083A	02-284	GSM362964	Tumor	low
LCS_076A	03-464	GSM362956	Tumor	high
LCS_139A	02-454	GSM363348	Tumor	high
LCS_198A	03-211	GSM363213	Tumor	low
LCS_041A	03-197	GSM363198	Tumor	high
LCS_119A	02-366	GSM363184	Tumor	high
LCS_101A	02-321	GSM362960	Tumor	high
LCS_008A	02-416	GSM363145	Tumor	high
LCS_067A	03-365	GSM363050	Tumor	low
LCS_029A	03-114	GSM362949	Tumor	high
LCS_346A	LCS-346	GSM363430	Tumor	high
LCS_064A	03-360	GSM363241	Tumor	low
LCS_241A	03-295	GSM363122	Tumor	low
LCS 096A	02-309	GSM362971	Tumor	low

Agilent_GSM	CGH_survival_group	Gender	Age	HBV viral status	ALT(>/<=50U/L)
		M	70	CC	low
		M	74	CC	low
		M	39	CC	low
		M	66	N	low
		M	54	N	low
		F	45	CC	low
		F	52	CC	low
		M	52	CC	low
		M	46	CC	low
		M	58	CC	low
		F	57	CC	low
GSM358056	G2	M	63	CC	low
		M	65	CC	low
GSM358055	G1	F	27	CC	low
		M	57	CC	low
		M	65	CC	low
		M	64	CC	low
		F	65	CC	low
GSM358045	G2	M	50	CC	low
GSM358062	G1	F	35	AVR-CC	low
		M	42	CC	low
		F	30	CC	low
		M	63	AVR-CC	low
		F	55	CC	low
		M	56	CC	low
		F	60	CC	low
GSM358083	G2	M	72	.	low
GSM358082	G1	M	61	CC	low
		M	72	CC	low
		M	53	CC	low
GSM358085	G2	M	43	.	low
		F	53	CC	low
		M	58	CC	low
GSM358079	G2	M	59	CC	low
		M	45	AVR-CC	low
		M	52	CC	low
		M	49	CC	low
GSM358098	G2	M	46	.	low
GSM358072	G1	M	46	CC	low
GSM358099	G1	M	50	.	low
GSM358033	G2	F	52	AVR-CC	low
		M	41	CC	low
		M	56	CC	low
		M	73	CC	low
GSM358058	G2	M	77	CC	low
		M	35	CC	low
		M	56	AVR-CC	low
GSM358053	G2	M	26	CC	low
		M	49	CC	low
GSM358084	G2	M	67	.	low

		M	54	AVR-CC	low
		M	53	CC	low
		F	47	AVR-CC	low
GSM358075	G2	M	49	CC	low
		M	43	AVR-CC	low
		M	53	N	low
		F	52	AVR-CC	low
		F	71	AVR-CC	low
		M	53	CC	low
		M	40	N	low
		M	56	AVR-CC	low
		M	39	CC	low
GSM358039	G1	M	61	CC	low
		M	49	CC	low
		F	30	CC	low
GSM358087	G2	M	76	.	low
GSM358034	G1	M	47	CC	low
		M	33	CC	low
		M	54	AVR-CC	low
		M	71	CC	low
		M	50	AVR-CC	low
		M	25	AVR-CC	low
		M	50	CC	low
GSM358086	G2	M	33	.	low
		M	71	CC	low
		F	50	CC	low
		F	48	CC	low
GSM358100	G1	F	60	.	low
		M	55	CC	low
		M	49	CC	low
		F	70	CC	low
		M	67	CC	low
		M	61	CC	low
		M	45	CC	low
		M	43	CC	low
		M	61	CC	low
		M	53	CC	low
		M	55	AVR-CC	low
		M	45	CC	low
		M	45	AVR-CC	low
GSM358041	G2	M	56	CC	low
		M	44	AVR-CC	low
		M	32	CC	low
		M	69	CC	low
		M	51	CC	low
		M	43	CC	low
		M	60	AVR-CC	low
		F	48	AVR-CC	low
		M	59	CC	low
		M	48	CC	low
GSM358050	G2	M	45	CC	low
		M	52	CC	low
		M	41	CC	low

		M	41	CC	low
		M	34	CC	low
GSM358067	G2	M	59	CC	low
		M	46	AVR-CC	low
		M	57	CC	low
GSM358046	G2	M	52	CC	low
		M	50	AVR-CC	low
		M	40	CC	low
		M	72	N	low
GSM358035	G2	M	63	CC	low
GSM358095	G2	M	55	.	low
		M	41	CC	low
		M	41	CC	low
		M	61	CC	low
		M	63	AVR-CC	low
		M	48	CC	low
		M	58	CC	low
GSM358077	G1	M	56	CC	low
		M	55	CC	low
		M	48	CC	low
		M	45	AVR-CC	low
		M	48	CC	low
		M	44	AVR-CC	low
		M	54	CC	low
		M	49	CC	low
		M	70	CC	low
GSM358060	G2	M	55	AVR-CC	low
		F	58	.	low
		M	50	CC	low
GSM358054	G2	M	50	CC	low
GSM358089	G1	M	41	.	low
GSM358078	G1	M	51	CC	low
		F	54	CC	low
		M	34	CC	low
		M	50	CC	low
		M	47	AVR-CC	low
		M	49	AVR-CC	low
		M	50	CC	low
		M	68	CC	low
		M	28	CC	high
		M	58	CC	high
GSM358043	G2	F	54	AVR-CC	high
GSM358032	G2	M	36	AVR-CC	high
		M	43	CC	high
GSM358097	G2	M	42	.	high
		M	43	AVR-CC	high
		M	44	AVR-CC	high
		M	66	.	high
GSM358029	G1	M	67	CC	high
		M	34	CC	high
GSM358071	G2	M	46	CC	high
		M	58	AVR-CC	high
		M	45	AVR-CC	high

		M	49	CC	high
		F	58	CC	high
		M	42	CC	high
GSM358036	G2	M	37	AVR-CC	high
		M	35	CC	high
		M	37	CC	high
		M	40	CC	high
GSM358047	G2	M	69	CC	high
		M	53	CC	high
		F	41	CC	high
		M	43	CC	high
		F	53	CC	high
GSM358063	G1	M	48	AVR-CC	high
GSM358049	G2	M	47	CC	high
		M	50	CC	high
		M	54	CC	high
		M	56	CC	high
		M	64	CC	high
		M	39	AVR-CC	high
		M	61	AVR-CC	high
		F	59	AVR-CC	high
		M	41	AVR-CC	high
		F	67	CC	high
GSM358081	G2	M	51	CC	high
		M	54	CC	high
GSM358042	G2	M	53	CC	high
		M	61	CC	high
GSM358074	G2	M	40	CC	high
		M	50	.	high
		F	49	AVR-CC	high
GSM358069	G2	M	58	CC	high
		F	69	CC	high
		M	50	CC	high
		M	39	CC	high
		M	35	AVR-CC	high
GSM358026	G2	M	47	CC	high
		M	63	CC	high
		M	58	CC	high
GSM358038	G2	M	53	CC	high
GSM358057	G2	M	50	CC	high
GSM358028	G1	M	32	CC	high
		M	59	AVR-CC	high
GSM358052	G1	M	41	CC	high
GSM358094	G1	M	48	.	high
		F	63	CC	high
GSM358031	G2	M	60	AVR-CC	high
		M	40	CC	high
		M	65	CC	high
		M	53	CC	high
		M	58	CC	high
		M	67	CC	high
		M	67	CC	high
		M	54	AVR-CC	high

		M	39	CC	high
GSM358037	G1	M	49	CC	high
		M	39	CC	high
		M	36	AVR-CC	high
		M	52	AVR-CC	high
		M	44	AVR-CC	high
		M	51	AVR-CC	high
GSM358096	G2	M	73	.	high
		M	48	CC	high
		M	50	CC	high
		M	41	AVR-CC	high
GSM358040	G2	M	34	CC	high
		M	44	CC	high
GSM358068	G2	M	35	CC	high
		M	62	CC	high
		M	45	AVR-CC	high
		M	54	AVR-CC	high
		M	47	CC	high
GSM358076	G2	M	59	AVR-CC	high
		M	50	CC	high
GSM358080	G2	M	33	AVR-CC	high
		M	41	CC	high
		M	50	N	high
GSM358051	G2	M	41	AVR-CC	high
		M	34	AVR-CC	high
		M	21	CC	high
		M	43	CC	high
GSM358073	G1	M	32	AVR-CC	high
GSM358044	G2	M	37	.	high
GSM358088	G2	M	47	.	high
GSM358070	G1	M	55	CC	high
		M	49	AVR-CC	high
		M	52	AVR-CC	high

Main Tumor Size (>/<=5 cm)	Multinodular	Cirrhosis	TNM staging	BCLC staging	CLIP staging
small	N	N	I	0	0
small	N	Y	I	A	0
small	N	N	I	0	0
large	N	Y	I	A	1
large	N	Y	I	A	0
small	N	Y	I	A	1
small	N	Y	II	A	0
large	N	Y	IIIC	C	0
large	N	Y	IIIA	C	4
small	Y	N	IIIC	C	1
small	N	Y	I	A	0
large	N	Y	I	C	3
large	Y	Y	II	A	2
small	N	N	I	A	1
large	N	N	II	A	0
large	N	Y	IIIB	A	1
small	N	Y	II	0	0
small	N	Y	I	A	0
large	Y	Y	III	B	2
small	N	Y	I	A	1
small	N	N	II	A	0
small	N	N	I	A	1
small	N	Y	I	A	1
large	N	Y	I	A	1
large	N	Y	IIIB	C	4
small	N	Y	II	A	1
small	N	Y	.	.	.
small	N	Y	I	A	1
large	N	Y	II	A	0
large	N	N	I	A	0
small	N	Y	.	.	.
small	N	Y	I	A	1
small	N	Y	II	A	0
large	N	Y	IIIB	A	1
.	N	Y	I	A	0
large	N	Y	IIIB	A	1
small	N	N	I	A	0
small	N	Y	.	.	.
small	N	Y	I	A	1
large	N	Y	.	.	.
small	N	Y	II	A	2
large	Y	Y	III	B	2
large	N	Y	II	A	0
large	N	Y	I	A	0
large	N	Y	II	A	0
large	Y	Y	IIIB	C	3
small	N	Y	I	A	0
small	N	Y	II	A	1
small	N	Y	II	A	0
small	N	Y	.	.	.

small	N	Y	I	A	0
small	N	Y	II	A	0
large	N	Y	II	A	0
large	N	Y	IIIB	C	5
large	N	Y	II	A	1
large	N	Y	IIIA	C	2
small	N	Y	I	0	1
small	N	Y	I	A	1
small	N	Y	II	A	0
small	N	Y	I	A	1
small	N	Y	I	A	1
large	N	N	I	A	1
small	N	Y	I	A	0
small	N	Y	I	A	0
small	N	N	I	A	0
large	N	Y	.	.	.
small	N	Y	IIIB	A	0
small	N	Y	I	A	0
large	N	Y	I	A	1
large	N	Y	II	A	0
large	Y	Y	IIIB	B	2
small	Y	Y	II	A	2
large	N	Y	I	A	0
large	Y	Y	.	.	.
small	N	Y	I	A	0
small	Y	Y	II	B	1
small	N	Y	II	0	0
small	N	Y	.	.	.
small	N	Y	II	A	1
large	N	Y	II	A	0
small	Y	Y	II	A	1
small	N	Y	I	A	0
small	N	Y	IIIA	C	2
small	N	Y	I	A	0
large	N	Y	I	A	1
large	N	Y	IIIA	C	2
large	N	Y	I	A	1
small	N	Y	II	0	0
small	N	Y	I	0	0
small	N	Y	II	A	0
small	N	Y	I	A	1
large	Y	Y	IIIA	B	1
large	N	Y	II	A	1
small	N	Y	I	A	1
large	Y	Y	IIIA	B	1
large	Y	Y	IIIA	B	2
small	N	Y	II	0	1
small	Y	N	II	A	2
small	N	Y	I	0	0
small	N	Y	I	A	0
small	N	Y	I	A	1
small	N	N	I	A	1
small	Y	Y	II	A	1

small	N	Y	II	A	0
small	N	Y	II	0	0
small	N	Y	I	A	0
small	Y	Y	IIIA	C	3
small	Y	Y	II	B	2
large	Y	Y	IIIA	B	2
large	Y	Y	IIIA	B	2
small	N	Y	I	0	1
small	N	Y	I	A	0
small	N	Y	I	A	0
small	N	Y	.	.	.
small	N	Y	II	A	1
large	N	Y	I	A	0
large	N	Y	I	A	0
large	N	Y	I	A	0
small	N	Y	I	0	1
small	N	Y	II	A	0
small	N	Y	II	A	0
small	N	Y	II	0	0
large	N	Y	II	A	1
small	N	N	I	0	0
large	N	Y	IIIB	A	3
small	Y	Y	II	A	2
small	Y	Y	II	A	1
small	N	Y	II	A	1
small	N	Y	I	A	1
small	N	Y	I	A	0
large	N	Y	IIIB	A	1
small	N	Y	I	A	0
small	N	Y	II	A	0
large	N	Y	.	.	.
large	N	Y	II	A	0
large	N	Y	IIIB	C	1
large	Y	Y	IIIA	C	3
small	N	Y	I	A	1
small	N	Y	I	A	0
small	N	N	II	A	0
small	N	Y	I	A	1
large	N	Y	II	A	1
small	Y	Y	II	B	2
small	N	Y	II	A	1
large	N	Y	I	A	1
small	N	Y	II	A	0
small	N	Y	II	A	1
small	Y	Y	.	.	.
small	N	Y	II	A	0
small	Y	Y	II	B	1
small	N	Y	.	.	.
large	Y	Y	IIIA	B	1
large	N	Y	IIIA	C	1
large	Y	Y	IIIA	C	2
small	N	Y	I	A	0
large	Y	Y	IIIA	B	2

large	Y	Y	IIIA	B	2
small	N	Y	I	0	0
small	N	Y	I	A	0
small	Y	Y	II	A	1
small	N	Y	IIIB	C	1
small	N	Y	I	A	2
large	N	Y	II	A	0
large	N	Y	I	A	1
large	N	Y	IIIA	C	2
large	N	Y	I	A	1
large	Y	Y	III	B	1
small	N	Y	II	A	0
large	N	Y	II	A	0
large	Y	Y	IIIA	C	3
small	N	Y	I	0	0
small	N	Y	I	A	0
small	N	Y	I	A	0
small	N	Y	II	A	2
large	N	Y	IIIA	C	2
small	N	Y	I	A	1
small	Y	Y	II	B	2
small	N	Y	II	A	0
small	Y	Y	IIIB	A	1
small	N	Y	II	A	1
small	N	Y	I	A	0
small	N	Y	I	A	0
large	Y	Y	IIIA	C	3
small	N	Y	I	A	0
large	N	Y	.	.	.
small	N	Y	II	0	1
small	Y	Y	II	B	1
small	N	Y	I	A	0
large	N	Y	II	A	1
small	N	Y	I	A	0
small	N	Y	II	A	0
small	Y	Y	IIIA	C	2
small	Y	Y	II	A	1
small	N	Y	IIIA	A	2
small	Y	Y	II	B	2
large	Y	Y	IIIA	C	2
small	N	Y	I	A	1
small	N	Y	I	A	0
small	N	Y	II	A	0
large	N	Y	.	.	.
small	N	Y	I	0	0
small	N	Y	II	A	0
large	N	Y	I	A	0
small	N	Y	II	A	1
large	N	Y	IIIA	C	2
small	N	Y	I	0	1
small	N	Y	II	A	0
large	N	Y	I	A	0
small	Y	Y	II	A	1

small	Y	Y	IIIC	C	3
large	N	Y	II	A	0
large	Y	Y	IIIA	C	4
small	N	Y	II	A	1
small	N	Y	I	A	0
small	Y	Y	II	B	2
large	N	Y	IIIB	A	0
large	Y	N	.	.	.
small	Y	Y	II	A	2
small	N	Y	I	A	0
small	N	Y	II	A	1
large	N	Y	IIIA	C	2
small	N	N	I	A	0
large	Y	Y	IIIB	C	3
small	N	Y	I	0	0
small	N	Y	I	A	1
large	N	Y	IIIC	C	0
large	N	Y	I	A	1
large	Y	Y	IIIA	B	2
small	N	Y	I	A	0
large	Y	Y	IIIA	B	1
small	N	Y	I	A	0
large	N	N	II	C	0
small	N	Y	I	A	0
large	Y	Y	IIIA	B	1
small	N	Y	II	A	1
small	N	N	I	A	0
small	Y	Y	II	B	2
small	Y	Y	.	.	.
small	N	Y	.	.	.
large	N	Y	I	A	1
small	N	Y	I	A	0
small	N	Y	I	A	2

AFP (>/<=300ng/ml)	Survival status	Survival months	Recurr status	Recurr months
high	0	58	0	58
low	0	66.6	0	66.6
low	0	67.3	0	67.3
high	1	10.4	1	10.4
low	0	52.8	0	52.8
high	0	60.8	0	60.8
low	0	38.7	0	38.7
low	0	28.7	0	28.7
high	1	12	1	12
low	0	54.5	0	54.5
low	0	57	0	57
high	0	54.9	0	54.9
high	0	56.3	0	56.3
high	0	55.2	0	55.2
low	0	66.6	0	66.6
high	0	59.5	1	59.5
low	0	57	0	57
low	0	50.7	0	50.7
high	1	30.9	1	30.9
high	0	54.7	0	54.7
low	0	57.3	1	41.6
high	0	65.1	0	65.1
high	1	36.4	1	36
high	0	7.3	0	7.3
high	1	3.4	1	1.5
high	0	59.2	0	59.2
high	1	7.3	1	0.2
high	0	54.3	0	54.3
low	0	61.8	0	61.8
low	0	14.4	0	14.4
low	0	63.8	0	63.8
high	0	65.5	0	65.5
low	0	52.8	1	20.8
high	0	53.8	0	53.8
high	0	62.4	0	62.4
high	0	24.9	0	24.9
low	0	10.4	0	10.4
low	1	7.3	1	5.3
high	0	54.2	0	54.2
high	0	52.1	0	52.1
high	1	27.5	1	27.5
high	0	32.8	0	32.8
low	1	28.2	1	28.2
low	0	52.3	0	52.3
low	0	19	0	19
high	1	13.8	1	13.8
low	0	53.2	0	53.2
high	1	23.9	1	23.9
low	0	51.4	0	51.4
low	0	51.6	0	51.6

low	0	61.2	0	61.2
low	1	5.8	1	5.8
low	1	8.8	1	8.8
high	1	2.3	1	2.3
high	1	16.5	1	16.5
high	1	14.6	1	14.6
high	0	51.1	0	51.1
high	1	7	1	7
low	1	9.1	1	6.2
high	0	54	1	18.5
high	0	56	0	56
high	0	59.7	0	59.7
low	0	60.7	1	32.7
low	1	27.1	1	10.2
low	1	45.9	1	45.9
high	1	3.8	1	3.8
low	1	57.9	1	57.9
low	0	60	1	6.7
high	1	12.6	1	2.5
low	0	36.6	1	36.6
low	1	12.7	1	8.4
high	0	62.6	0	62.6
low	1	12.6	1	12.6
high	1	1.8	1	1.8
high	0	53.3	0	53.3
low	0	58.4	1	23.6
low	1	32.6	1	32.6
low	0	51.7	0	51.7
high	1	22.2	1	14.6
low	1	37.9	1	37.9
low	0	67.1	0	67.1
.	0	53.5	0	53.5
high	1	7.5	1	4.4
low	0	59.4	0	59.4
high	0	62.9	0	62.9
high	1	10	1	1.9
high	1	53.3	1	53.3
low	0	64.5	0	64.5
low	0	66	0	66
low	1	7.6	1	7.6
high	0	60.3	1	35.2
low	0	2	0	2
high	0	65.4	0	65.4
low	0	55.8	0	55.8
low	0	53.8	0	53.8
high	0	20.6	0	20.6
high	0	56.9	1	21.3
high	0	57.1	0	57.1
low	0	53.3	0	53.3
low	0	51.3	0	51.3
high	0	57.5	1	30.1
high	0	53.6	1	40.1
low	0	65.1	0	65.1

low	1	15.1	1	5.8
low	1	53	1	53
low	1	35.9	1	29.9
high	1	4	1	4
high	1	17.6	1	9.1
high	1	47.1	1	3.2
high	1	20.3	1	20.3
high	0	52.7	0	52.7
low	0	51.1	0	51.1
low	1	8	1	8
low	0	56.6	0	56.6
high	1	26.5	1	21.8
low	0	66.3	0	66.3
low	1	4.5	1	4.5
high	1	12	1	12
high	0	18.6	0	18.6
low	0	55.6	1	19.1
low	0	53.9	1	9.4
low	0	57.3	1	21.5
high	1	13.5	1	13.5
low	0	53.3	0	53.3
high	1	15.2	1	3.9
high	0	60.5	0	60.5
low	1	47.9	1	13.4
high	0	54.8	1	54.8
high	0	57.9	1	13.1
low	1	52.7	1	12.4
high	0	3	0	3
low	0	52.7	0	52.7
low	0	55.2	0	55.2
low	1	34.4	1	29.1
low	0	53.8	0	53.8
low	1	18	1	18
high	1	3.5	1	3.2
high	0	56.3	0	56.3
low	1	31.9	1	13.3
low	0	57.9	1	30.7
high	0	3.8	1	3.8
high	1	3.3	1	3.3
high	1	46.1	1	46.1
high	1	7.7	1	7.7
high	1	36.5	1	28.4
low	1	30.1	1	10
high	0	65.4	0	65.4
high	1	4.6	1	1.3
low	1	51.6	1	51.6
low	1	28.7	1	28.7
high	1	15.4	1	11
low	1	59.2	1	50
low	1	5.2	1	0.1
low	1	14.3	1	14.3
low	0	66.1	0	66.1
high	0	39.4	0	39.4

high	1	54.8	1	46.3
low	0	57.3	1	11.6
low	0	57.7	1	51.1
low	0	61.4	1	8.8
low	1	2.5	1	1.5
high	1	37.2	0	37.2
low	0	4.8	0	4.8
high	0	57.5	1	4.7
high	1	14.1	1	8.4
.	0	64.3	0	64.3
low	1	17.8	1	17.8
low	0	55.3	0	55.3
low	0	42.7	0	42.7
high	0	57.5	0	57.5
low	0	52.2	0	52.2
low	1	50.1	1	43.2
low	0	58	0	58
high	0	52.4	1	11.8
high	1	3	1	3
low	1	28.8	1	28.8
high	1	19.2	1	19.2
low	1	23.5	1	23.5
low	0	59.8	0	59.8
high	1	16.2	1	5.1
low	0	61.5	1	27.9
low	0	59.9	0	59.9
high	1	8.9	1	8.9
low	0	5.5	1	1.4
high	0	57	0	57
high	0	65.4	0	65.4
low	1	21.3	1	21.3
low	0	66.3	0	66.3
high	0	58.9	0	58.9
low	0	64.6	0	64.6
low	0	57.3	1	46.3
low	1	19.6	1	19.6
low	1	60.5	1	22.8
high	1	4.5	1	4.5
high	1	26.9	1	26.9
low	0	54.9	0	54.9
high	0	64.2	0	64.2
low	0	48.4	1	49.1
low	0	57.7	0	57.7
high	1	3.8	1	0.8
low	0	54.7	0	54.7
low	0	60.4	1	57.7
low	0	56.1	1	24.6
low	1	32.7	1	4.6
high	1	7.8	1	5.2
high	0	62.8	1	8
low	1	6.7	1	6.7
low	0	62.6	1	48
low	1	42.2	1	6.8

high	0	51.2	0	51.2
low	0	48.8	0	48.8
high	1	8.3	1	8.3
low	0	9.5	1	9.5
low	0	60.1	0	60.1
high	0	4.5	0	4.5
low	1	14.3	1	7.3
high	0	55.5	1	20
high	0	67.1	0	67.1
low	0	67	1	51.1
low	0	4.8	1	2.7
high	1	13.6	1	1.7
low	0	52.9	0	52.9
high	1	23	1	23
low	0	61.5	0	61.5
high	1	9.5	1	9.5
low	0	60.4	1	19.4
.	0	62	0	62
high	0	54.2	1	29.9
low	0	67.4	0	67.4
.	1	13.3	1	13.3
low	0	14.3	1	4.2
low	0	57.6	1	2.9
low	0	57.9	0	57.9
low	0	16.3	0	16.3
high	0	66.1	0	66.1
low	1	33	1	26.4
high	0	54.2	1	2.8
low	1	18.2	1	18.2
high	1	3.7	1	3.7
high	0	54.2	0	54.2
low	0	55.4	0	55.4
high	1	48.1	1	40.4
