# Supplementary material for: M6A Modifier-Mediated Methylation Characterized by Diverse Prognosis, Tumor Microenvironment, and Immunotherapy Response in Hepatocellular Carcinoma
Source: J Oncol. 2022 Aug 16;2022:2513813. doi: 10.1155/2022/2513813 (PMC9398803; doi:10.1155/2022/2513813)
Supplement: Supplementary Materials — Supplementary Figure 1. Consensus clustering analyses of stratifying HCC cases in TCGA cohort into three m6A methylation patterns according to 23 m6A regulators. (A) Heatmap for the consensus matrix k = 3. (B) Cumulative distribution function (CDF) under diverse k values. (C) Delta area diagram for relative alterations in area under CDF curves. (D) The tracking plot for HCC samples under different k values. Supplementary Figure 2. Consensus clustering analyses for clustering three m6A genomic phenotypes in the light of the expression profiling of m6A-associated genes in TCGA cohort. (A) Heatmap for the consensus matrix k = 3. (B) CDF under diverse k values. (C) Delta area diagram for relative alterations in area under CDF curves. (D) The tracking plot for HCC samples under different k values. Supplementary Figure 3. Subgroup analysis of the prognosis value of m6A score among HCC patients in TCGA data set. Kaplan-Meir curves of cases with high or low m6A score in each subgroup: (A) age ≥ 65; (B) age < 65; (C) female; (D) male; (E) G1-2; (F) G3-4; (G) stage I-II; (H) stage III-IV. P values were determined through log-rank tests. Supplementary Table 1. The clinical information of HCC samples in the TCGA data set. Supplementary Table 2. The clinical information of HCC samples in the GSE14520 data set. Supplementary Table 3. The list of 331 m6A phenotype-associated DEGs. [file 2513813.f1.zip › 2513813.f1/Supplementary table 3.pdf]

Supplementary table 3. The list of 331 m6A phenotype-associated DEGs.
IGF2BP2
LRRC1
IGF2BP1
MYBL2
AURKB
UBE2C
BAZ2A
HSD11B1
GLYAT
INCENP
NUP205
TTC36
CYP8B1
CDC20
SLC27A5
KCTD17
BEX2
H2AFY2
SART3
NCOA6
MSI1
FAM208A
NAT2
ADH1B
MAU2
AFP
ACSM5
PTTG1
CD24
CRLF3
NEMP1
TAT
ETNPPL
TOPBP1
LINC00844
GAL3ST1
WSB1
CENPM
ARL6IP6
DUSP9
SINHCAF
ACTL6A
CES2
DIP2B
AQP9
CAND1
LRIG3
GLYATL1
ZDHHC17
VEGFB
ADM2
MIB1

TMPO
ACSM2A
HID1
PRRC2C
UTRN
VHL
DHX9
MEX3C
XPO1
TPR
WAC
MOGAT2
SPIN1
SPHK1
ADD3
CAPRIN1
KIF2A
MAL2
SETD5
C8A
CHD6
FAM241B
CTDSPL2
SMC4
CDK2
MISP
CIB2
MLLT10
TRNP1
SLC10A1
ACLY
SMAD5
SUZ12
CCDC93
DIDO1
BTBD3
C6
DCAF7
ZNF710
HACD2
BCOR
MAML1
MED12
TOP2B
AL390728.4
CEP192
STK24
SMC3
NUP160
ASPDH
EIF4G2
ADH1C
FUBP1

TRRAP
C3P1
PRAME
PPP1R12A
ACSM2B
AC016735.1
SRPK1
CFHR4
WDFY1
UPK3A
CAPG
H19
DHX15
THOC2
RNF44
SMCHD1
PRRC2A
AC112206.2
SUGP2
DMTF1
ADCY6
BRD1
SPINT1
GPC3
SUPT20H
MDK
HMGXB4
SP3
LARP1
GCC2
MOB1A
KDM3B
TNRC18
PTPN12
SLC28A1
HEATR1
ALDH1L1
RAB34
HSD17B6
LINC01093
AC132008.2
DMKN
ADAM17
MFSD2A
CTDSP2
PDE9A
USP1
WNK1
MIS18BP1
PRPF38B
LSM14B
RTP3
SLC22A1

IPO7
CYP4F2
MFSD14B
VEZF1
LDB1
APPL2
EPB41L2
CCL20
AC008735.2
ASAP1
HAO1
TRIM31
PCK1
KDM6A
LINC01554
MT1X
THRSP
PAPSS1
APOA5
SMC2
XPOT
TARBP1
ZMIZ1
TUBA1B
AC137723.1
CEP170
FAM168B
NCAPD3
ZNF384
CCND2P1
ETV4
ADAM10
RDH16
EML4
ZDHHC20
ANXA10
NID1
PI4KA
WEE1
IARS
UGT2B10
RAVER1
ATP11A
HLTF
WDR6
NPEPPS
S100A14
BEX3
FXYD3
SRSF11
FNBP1L
2-Sep
F9

CCDC88A
FABP4
CDC42SE1
GMCL1
NDE1
PIP5K1A
CYP2C8
SLC39A6
UGT2B15
PNISR
RNU6-850P
CYP2A6
CDK2AP1
MAT2A
NKTR
EPS8L3
AC113191.1
ANKRD10-IT1
TFDP1
ANXA13
MLLT6
PIAS3
ACVR1
LAMC1
ZNF217
C4BPA
LENG8
RERE
UBD
UGT2B7
MAP4K3
RFC3
PLPP2
CDC14B
COL5A2
CALU
FARP1
DEK
HOOK1
ADAM9
MIR647
RAB11FIP1
MIR4292
AASS
ZNF687
TAF6
BCL9L
SUSD4
ARHGEF11
DDX39B
FXYD2
ZNF189
APOC1

MPDZ
INTS3
SULT2A1
DAPK1
ATP11C
LIMA1
DLK1
ERMP1
IL1RAP
MPZL2
VNN2
CYP4A22
GLUD2
SPARCL1
TFRC
IQGAP1
ST5
F12
MMP9
LGR4
CYP2A7
NFKBIZ
HIF1A
AC021074.2
DLG5
RPS6KA3
SLC4A4
HLF
FETUB
ALAD
PHLDB1
TMEM150C
AC005336.1
OAS3
JAG1
AMACR
SKAP2
AC026403.1
LPCAT3
UGT1A1
SELENOP
SLC25A15
ENC1
PFKFB3
SLC25A25
SCRN1
MIR621
SLCO1B1
AC108751.5
LTBP1
AL451060.1
C4A
ACSM3

SORD2P
C4B
MIR1295A
PHYHIPL
MBNL3
MTND4P20
RNU1-70P
SLC6A1
UGT1A9
GLUL
COL3A1
APOC1P1
TBX3
SNCG
