# Supplementary figures and images for: M6A Modifier-Mediated Methylation Characterized by Diverse Prognosis, Tumor Microenvironment, and Immunotherapy Response in Hepatocellular Carcinoma
Source: J Oncol. 2022 Aug 16;2022:2513813. doi: 10.1155/2022/2513813 (PMC9398803; doi:10.1155/2022/2513813)

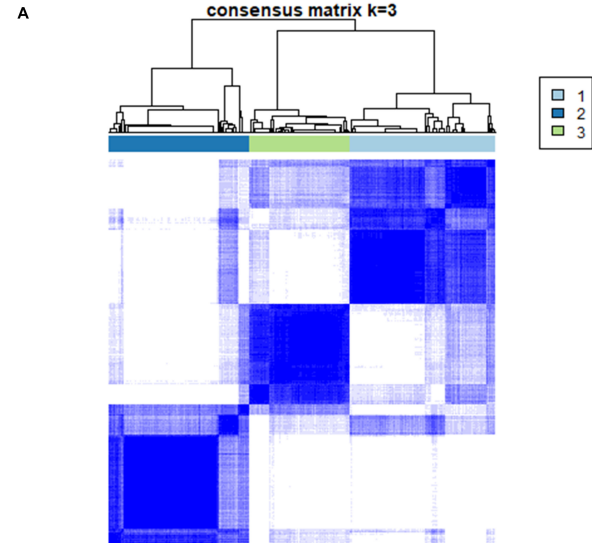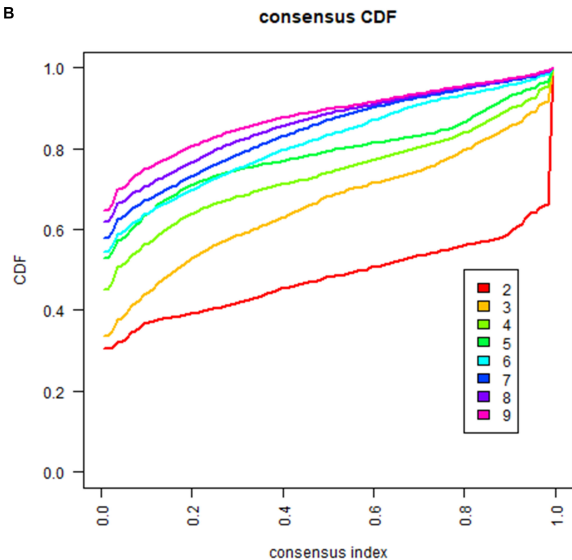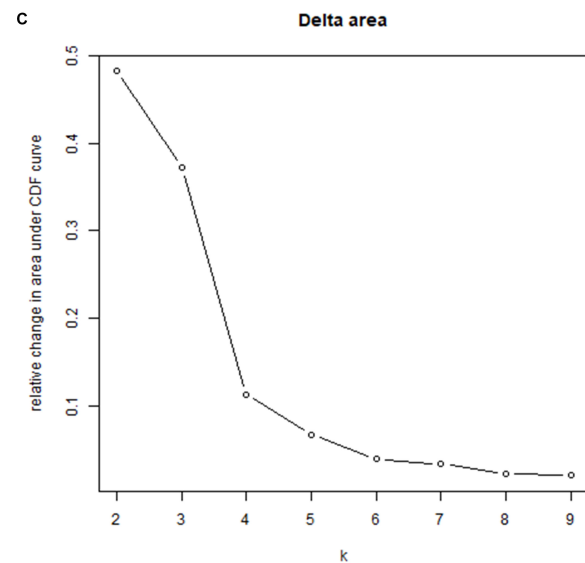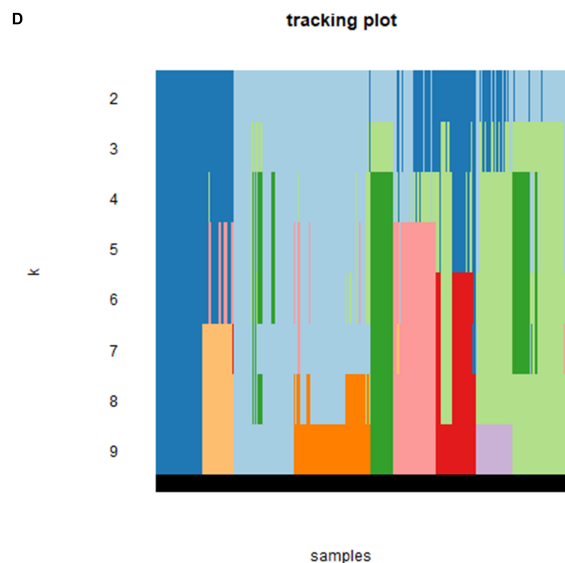

Supplement: Supplementary Materials — Supplementary Figure 1. Consensus clustering analyses of stratifying HCC cases in TCGA cohort into three m6A methylation patterns according to 23 m6A regulators. (A) Heatmap for the consensus matrix k = 3. (B) Cumulative distribution function (CDF) under diverse k values. (C) Delta area diagram for relative alterations in area under CDF curves. (D) The tracking plot for HCC samples under different k values. Supplementary Figure 2. Consensus clustering analyses for clustering three m6A genomic phenotypes in the light of the expression profiling of m6A-associated genes in TCGA cohort. (A) Heatmap for the consensus matrix k = 3. (B) CDF under diverse k values. (C) Delta area diagram for relative alterations in area under CDF curves. (D) The tracking plot for HCC samples under different k values. Supplementary Figure 3. Subgroup analysis of the prognosis value of m6A score among HCC patients in TCGA data set. Kaplan-Meir curves of cases with high or low m6A score in each subgroup: (A) age ≥ 65; (B) age < 65; (C) female; (D) male; (E) G1-2; (F) G3-4; (G) stage I-II; (H) stage III-IV. P values were determined through log-rank tests. Supplementary Table 1. The clinical information of HCC samples in the TCGA data set. Supplementary Table 2. The clinical information of HCC samples in the GSE14520 data set. Supplementary Table 3. The list of 331 m6A phenotype-associated DEGs. [file 2513813.f1.zip › 2513813.f1/Supplementary figure 1.pdf]

A

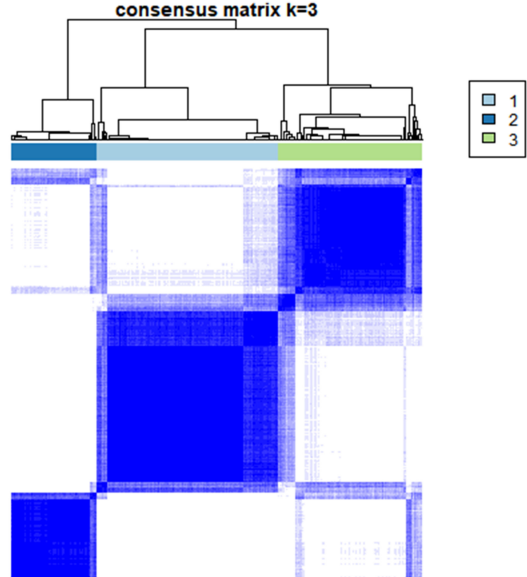

B

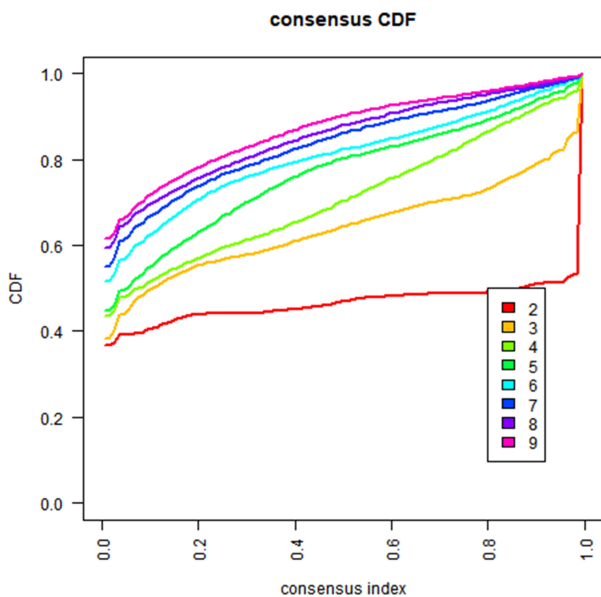

C

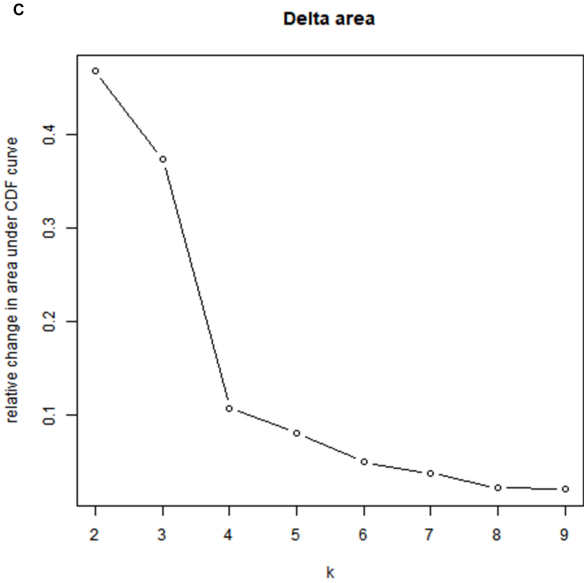

D

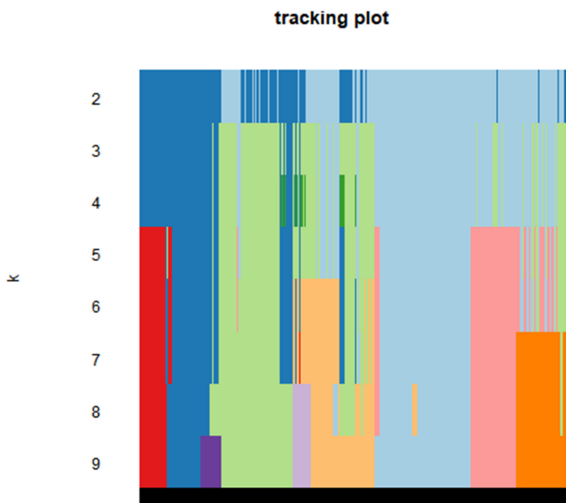

Supplement: Supplementary Materials — Supplementary Figure 1. Consensus clustering analyses of stratifying HCC cases in TCGA cohort into three m6A methylation patterns according to 23 m6A regulators. (A) Heatmap for the consensus matrix k = 3. (B) Cumulative distribution function (CDF) under diverse k values. (C) Delta area diagram for relative alterations in area under CDF curves. (D) The tracking plot for HCC samples under different k values. Supplementary Figure 2. Consensus clustering analyses for clustering three m6A genomic phenotypes in the light of the expression profiling of m6A-associated genes in TCGA cohort. (A) Heatmap for the consensus matrix k = 3. (B) CDF under diverse k values. (C) Delta area diagram for relative alterations in area under CDF curves. (D) The tracking plot for HCC samples under different k values. Supplementary Figure 3. Subgroup analysis of the prognosis value of m6A score among HCC patients in TCGA data set. Kaplan-Meir curves of cases with high or low m6A score in each subgroup: (A) age ≥ 65; (B) age < 65; (C) female; (D) male; (E) G1-2; (F) G3-4; (G) stage I-II; (H) stage III-IV. P values were determined through log-rank tests. Supplementary Table 1. The clinical information of HCC samples in the TCGA data set. Supplementary Table 2. The clinical information of HCC samples in the GSE14520 data set. Supplementary Table 3. The list of 331 m6A phenotype-associated DEGs. [file 2513813.f1.zip › 2513813.f1/Supplementary figure 2.pdf]

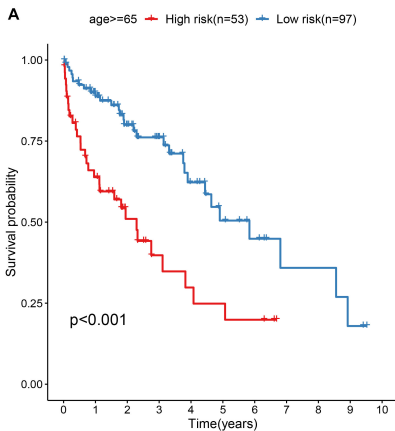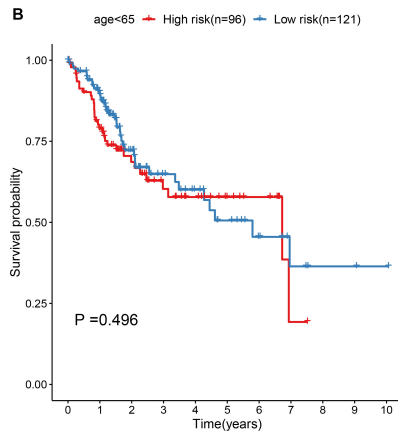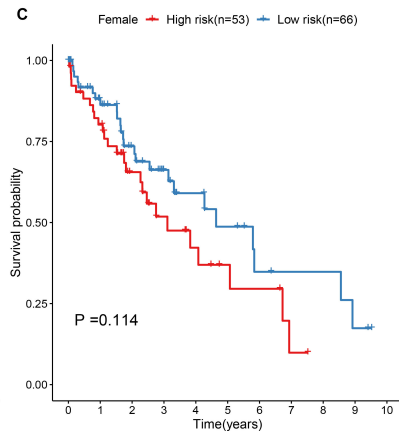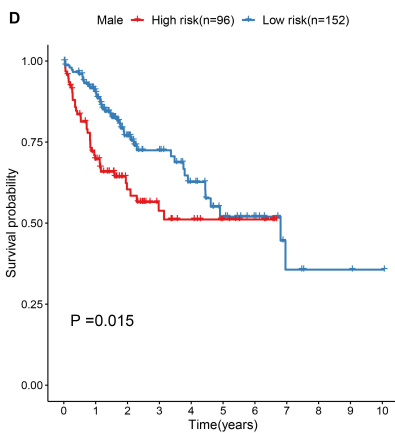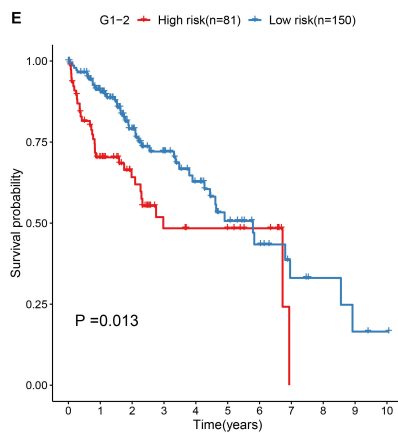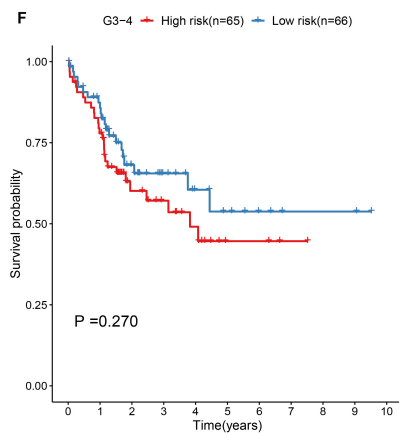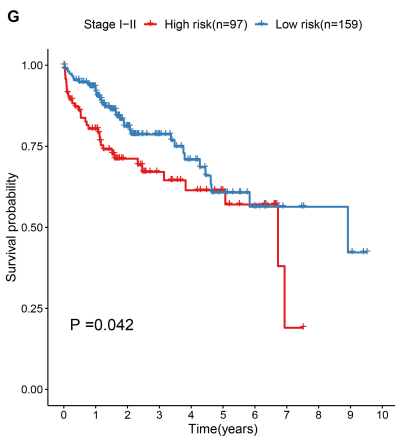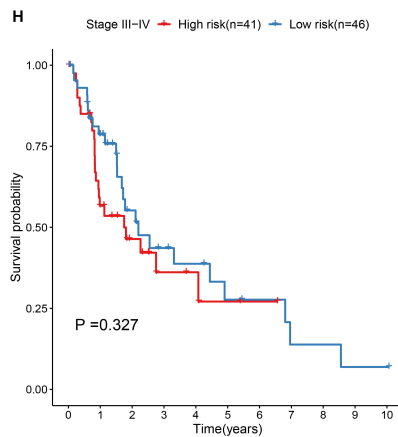

Supplement: Supplementary Materials — Supplementary Figure 1. Consensus clustering analyses of stratifying HCC cases in TCGA cohort into three m6A methylation patterns according to 23 m6A regulators. (A) Heatmap for the consensus matrix k = 3. (B) Cumulative distribution function (CDF) under diverse k values. (C) Delta area diagram for relative alterations in area under CDF curves. (D) The tracking plot for HCC samples under different k values. Supplementary Figure 2. Consensus clustering analyses for clustering three m6A genomic phenotypes in the light of the expression profiling of m6A-associated genes in TCGA cohort. (A) Heatmap for the consensus matrix k = 3. (B) CDF under diverse k values. (C) Delta area diagram for relative alterations in area under CDF curves. (D) The tracking plot for HCC samples under different k values. Supplementary Figure 3. Subgroup analysis of the prognosis value of m6A score among HCC patients in TCGA data set. Kaplan-Meir curves of cases with high or low m6A score in each subgroup: (A) age ≥ 65; (B) age < 65; (C) female; (D) male; (E) G1-2; (F) G3-4; (G) stage I-II; (H) stage III-IV. P values were determined through log-rank tests. Supplementary Table 1. The clinical information of HCC samples in the TCGA data set. Supplementary Table 2. The clinical information of HCC samples in the GSE14520 data set. Supplementary Table 3. The list of 331 m6A phenotype-associated DEGs. [file 2513813.f1.zip › 2513813.f1/Supplementary figure 3.pdf]
